# A Homoleptic Copper(I) Acetylene Complex

**DOI:** 10.1002/chem.71597

**Published:** 2026-08-21

**Authors:** Julie Willrett, Elisa Zöllner, Burkhard Butschke, Ingo Krossing

**Affiliations:** ^1^ Institut Für Anorganische und Analytische Chemie and Freiburger Materialforschungszentrum (FMF) Albert‐Ludwigs‐Universität Freiburg Freiburg Germany

**Keywords:** alkyne ligands, copper, quantum chemical calculations, weakly coordinating anions, x‐ray diffraction

## Abstract

We report the synthesis and characterization of the Cu(I) complex [Cu(C_2_H_2_)_3_][Al(OR^F^)_4_] (**1**), which represents a very rare example of a homoleptic acetylene complex. **1** was synthesized from the dinitrogen complex [(N_2_)Cu{Al(OR^F^)_4_}] by simple ligand substitution. While the crystallographic description of **1** is complicated by an incommensurate modulated structure, the molecular connectivity could be unambiguously determined, and the identity of **1** was further confirmed by NMR and Raman spectroscopy as well as extensive quantum chemical calculations.

## Graphical abstract

Ligand substitution of weakly coordinated N_2_ in [(N_2_)Cu{Al(OR^F^)_4_}] (R^F^ = C(CF_3_)_3_) leads to the homoleptic acetylene complex [Cu(C_2_H_2_)_3_]^+^[Al(OR^F^)_4_]^−^, which was characterized by single crystal x‐ray diffraction, NMR, and Raman spectroscopy, as well as by extensive quantum chemical calculations.

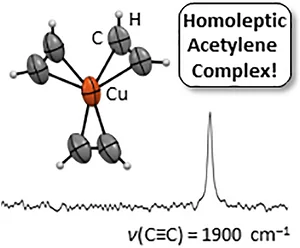

## 1 Introduction

Acetylene (C_2_H_2_, systematically named ethyne) is the simplest alkyne and a useful synthetic building block for more complex organic molecules [1], and it is also widely used as a welding gas [2]. Industrially, C_2_H_2_ is produced either from coal via calcium carbide as an intermediate or by hydrocarbon cracking [1]. In its purified form and at pressures above 2 bar, acetylene exhibits a high tendency to explode violently [3]. Consequently, commercial C_2_H_2_ is typically sold and stored in gas cylinders filled with a porous material and acetone, which acts as a solvent and stabilizer [3]. Particular caution is required when working with acetylene in the presence of copper as the formation of explosive acetylides is known from elemental copper and copper alloys as well as from various Cu(I) and Cu(II) sources in basic media [4].

Despite the growing interest in the synthesis of coinage metal acetylene complexes over the last years, only a limited number of structurally characterized examples are reported in the literature (Figure 1A). Most of these complexes feature a monocationic Cu(I) or Ag(I) atom supported by a strongly coordinating, polydentate auxiliary ligand and a single acetylene ligand [5, 6, 7, 8, 9, 10]. Only recently, the first example of a Au(I) acetylene complex was reported, which was prepared *in crystallo* from the corresponding carbonyl complex [11]. Due to the strongly donating auxiliary ligands in these examples, the C≡C stretching vibrational frequencies of the acetylene ligand are strongly red‐shifted compared to free acetylene (1974 cm^−1^) [12]. This behavior can be rationalized using the Dewar–Chatt–Duncanson (DCD) model, originally developed for metal olefin complexes [13], which is also applicable to metal acetylene complexes [14]. According to the DCD model, the metal‐acetylene bond can be described by the synergistic *σ*‐donation from the acetylene HOMO in an unoccupied metal orbital and the *π*‐backdonation from the metal into the acetylene LUMO. Both interactions weaken the C≡C triple bond, leading to a red‐shift in the stretching frequency. Notably, the *π*‐backdonation is enhanced by strongly donating additional ligands. As an approximation, the magnitude of this red‐shift can serve as a measure of the metal acetylene bond strength [14].

**FIGURE 1:**
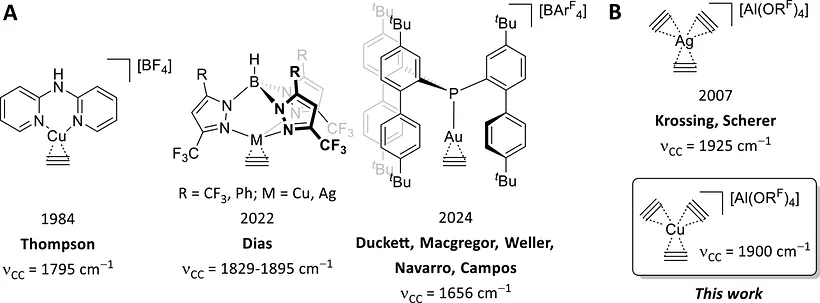
Examples for A) heteroleptic coinage metal acetylene complexes and B) homoleptic coinage metal acetylene complexes (with their acetylene C≡C stretching frequencies) [5, 10, 11, 15]. Ar^F^ = 3,5‐(CF_3_)_2_C_6_H_3_, R^F^ = C(CF_3_)_3_.

While there are multiple examples for heteroleptic coinage metal acetylene complexes, only two structurally analysed homoleptic acetylene complexes have been reported (Figure 1B): the Ag(I) complexes [Ag(C_2_H_2_)_3‐4_][Al(OR^F^)_4_] (R^F^ = C(CF_3_)_3_), where the number of acetylene ligands depends on the reaction stoichiometry [15]. In these complexes, acetylene is very weakly bound, as reflected by the high C≡C stretching frequency, close to that of free acetylene. Although homoleptic acetylene complexes have been observed for all coinage metals in the gas phase [16, 17, 18], homoleptic Cu(I) and Au(I) complexes have not been isolated in the condensed phase up to now. Attempts to generate [Au(C_2_H_2_)*_n_*]^+^ from the precursor [Au(CO)_2_]^+^ resulted only in decomposition, even at −35°C [19]. The preparation of a homoleptic Cu(I) complex has long been hindered due to the lack of a suitable Cu(I) precursor.

In 2024, our group reported the facile synthesis of the very weakly coordinated Cu(I) dinitrogen complex [(N_2_)Cu{Al(OR^F^)_4_}] [20], which has already been employed in the successful synthesis of Cu(I) complexes with extremely weakly coordinating arenes and high Cu^+^/Cu^0^ redox potentials up to 1.5 V vs. ferrocenium/ferrocene [21]. Inspired by related work by Peters et al., who employed a heteroleptic Fe(II) dinitrogen complex as a precursor for acetylene coordination via ligand substitution [22], we utilized [(N_2_)Cu{Al(OR^F^)_4_}] as a synthon for a homoleptic Cu(I) acetylene complex. The resulting compound was characterized by single crystal x‐ray diffractometry (scXRD), as well as NMR and vibrational spectroscopy.

## 2 Results and Discussion

A solution of [(N_2_)Cu{Al(OR^F^)_4_}] in pentafluorobenzene (5FB) was frozen, evacuated and subsequently warmed to room temperature to obtain the reaction mixture under the vapour pressure of the solvent. For safety reasons, acetylene was then added using a bubbler, while always allowing for pressure equalization. After acetylene addition, the reaction mixture was stirred at −40°C for ten minutes and was then layered with *n*‐pentane to yield colourless crystals (Equation (1)). It is crucial to maintain low temperatures at all times during the layering and crystallization process. When handled at room temperature, a dark solid starts to form within 30‐60 min, and a copper mirror becomes visible on the glass wall of the Schlenk tube. Under an argon atmosphere and when stored at −40°C, the crystals are stable for up to two weeks before decomposition becomes apparent.

(1)

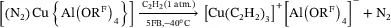




Single crystal x‐ray diffractometry (scXRD) of the colourless crystals revealed the structure of the homoleptic Cu(I) acetylene complex [Cu(C_2_H_2_)_3_][Al(OR^F^)_4_] (**1**, Figure 2A). The crystals were selected and mounted at −40°C. At this temperature, decomposition of the crystals takes ca. 20 min under perfluoropolyether oil. At room temperature, the crystals immediately start to decompose. This decomposition becomes visible in a colour change of the formerly colourless crystals to a copper colour. The limited stability of **1** can be explained to two factors: the high redox potentials reported for similar Cu(I) complexes [21], explaining the formation of the copper mirror, and possibly the formation of insoluble copper acetylides [23] or oligo‐/polyacetylene formed by oxidative coupling [24], causing the dark solid.

**FIGURE 2:**
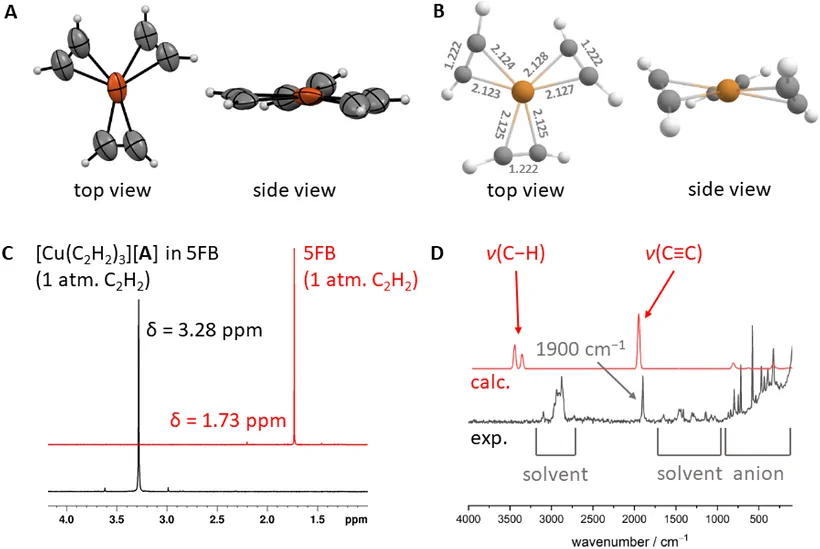
(A) Molecular structure of the cationic moiety of [Cu(C_2_H_2_)_3_][Al(OR^F^)_4_] (1, solved in P2_1_, R_1_ = 11.62 %, wR_2_ = 31.76 %). Thermal ellipsoids are shown at the 50 % probability level.  The structure is incommensurately modulated (see section S4), causing the high R‐values. The full molecular structure of 1 with anion and disorders included is shown in Figure S7. B) Computed ground state structure and bond lengths (in Å) of the cationic moiety of 1 at the RI‐r^2^SCAN‐3c(D4)/def2‐mTZVPP level of theory with the (approximate) point group D_3_. C) Excerpt from the ^1^H NMR spectra (300.18 MHz, 5FB under 1 atm. C_2_H_2_, room temperature) of 1 (black) and a blind sample of C_2_H_2_ (1 atm.) in 5FB (red). A = Al(OR^F^)_4_. D) Experimental (black) Raman spectrum of crystals of 1, embedded in a solvent mixture of 5FB and n‐pentane. A reference spectrum for the assignment of solvent bands is included in the Figure S6. Computed (RI‐r^2^SCAN‐3c(D4)/def2‐mTZVPP, red) Raman spectrum of [Cu(C_2_H_2_)_3_]^+^.

In the solid state, **1** consists of separated cations and anions as opposed to the ion‐paired precursor [(N_2_)Cu{Al(OR^F^)_4_}] [20]. This difference reflects the higher coordinative strength of acetylene relative to dinitrogen. While the identity of **1** could be determined without doubt, treatment of the scXRD data of all the many data sets recorded proved challenging. In all tested crystals, an incommensurate modulation of the crystal structure led to ambiguities in the space group determination, as well as to a model, which refined only to poor *R*_1_‐, w*R*_2_‐ and GoF‐values (for a detailed discussion of the treatment of the crystallographic data, see section S4). In our solution of the structure in the monoclinic space group *P*2_1_, the entire cationic moiety of **1** is disordered over two positions with occupancies of approximately 0.5:0.5. In the analogous silver complex, only one acetylene ligand is disordered, but the position of the silver atom is fixed. In **1**, even the copper atom occupies two distinct sites (see Figure S9). We suspect this ‘blurred’ cation position in the crystal lattice to be the origin of the observed structure modulation. The crystal structure is mainly defined by the anion positions, leaving a lot of space and mobility for the much smaller cations. Due to this cation disorder, the restraints necessary for structural refinement and the overall data quality, we renounce from a detailed discussion of bond lengths and angles in **1**. The cationic moiety of **1** adopts an approximate *D*_3_ symmetry, which is reported to be the preferred ground state symmetry for [Cu(C_2_H_2_)_3_]^+^ [16]. This structure also matches our optimized structure using density functional theory (Figure 2B). In the analogous silver complex [Ag(C_2_H_2_)_3_][Al(OR^F^)_4_], the cationic moiety possesses *C*_2_ symmetry, which was explained by H∙∙∙F contacts with the anion in the crystal [15]. Intriguingly, for [Ag(C_2_H_2_)_3_]^+^, the *C*_2_ and the *D*_3_ gas‐phase structures are almost isoenergetic [15, 25]. By contrast, structure optimization calculations of [Cu(C_2_H_2_)_3_]^+^ reliably yield the (approximate) *D*_3_ structure, and only when enforced, the *C*_2_ structure is obtained as a local minimum situated 30 kJ mol^−1^ higher in energy (DLPNO‐CCSD(T)/def2‐QZVPP//RIJCOSX‐B3LYP(D4)/def2‐TZVPP level of theory).

NMR spectra of **1** were recorded immediately after preparation under an acetylene atmosphere, and we were able to obtain spectra at room temperature showing minimal decomposition as most decomposition products are not soluble in 5FB. After the measurements, a small amount of a dark solid was visible in the NMR tube. Under an argon atmosphere, more pronounced anion decomposition is evident in the ^19^F and ^27^Al NMR spectra. In the ^1^H NMR spectrum of **1**, only one singlet is visible at 3.28 ppm corresponding to a coalescence signal of **1** with the acetylene atmosphere. Compared to a blind sample of acetylene in 5FB, this value represents a clear downfield shift of around 1.5 ppm (Figure 2C, computed downfield shift without coalescence: 2.6 ppm, RIJCOSX‐B3LYP(D4)/def2‐TZVPP). Notably, this shift is more pronounced than in [Ag(C_2_H_2_)_3_][Al(OR^F^)_4_] (δ = 2.87 ppm) [15], indicating a stronger de‐shielding of the acetylene protons in **1** and thus a stronger copper‐acetylene interaction. In the ^13^C NMR spectrum, a significant shift relative to free acetylene or [Ag(C_2_H_2_)_3_][Al(OR^F^)_4_] was not observed (computed shift without coalescence: 9 ppm, RIJCOSX‐B3LYP(D4)/def2‐TZVPP).

The Raman spectrum of crystals of **1** (Figure 2D) shows one strong band for the acetylene C≡C stretching vibration at 1900 cm^−1^ (full assignment of Raman data in Table S1). Compared to free acetylene, this value represents a red‐shift of 74 cm^−1^. Hence, this band is slightly more red‐shifted than the corresponding band in [Ag(C_2_H_2_)_3_][Al(OR^F^)_4_] (1925 cm^−1^) [15], indicating that the acetylene ligands in **1** are bound more strongly.

To confirm these observations, quantum chemical calculations were performed to determine complexation energies for Cu^+^ and Ag^+^ with *n* C_2_H_2_ ligands (Table 1). For the calculations, the very cost‐effective functional r^2^SCAN‐3c [26], the commonly used functional B3LYP [27, 28, 29] and the ‘gold standard’ for quantum chemical calculations, coupled cluster (CC) theory [30, 31] were used. While B3LYP provides closer agreement with the CC results than r^2^SCAN‐3c, all methods yield consistent trends. The first and the second acetylene ligand bind significantly stronger to Cu^+^ than to Ag^+^, whereas the third ligand exhibits similar binding energies for both metals. The complexation of a fourth ligand, however, is endergonic for Cu^+^ and slightly exergonic for Ag^+^. This observation explains why the isolation [Ag(C_2_H_2_)_4_][Al(OR^F^)_4_] was possible at very low temperatures [15], while the formation of the Cu(I) *tetra*‐acetylene complex was not observed, even though an excess of C_2_H_2_ was used in each reaction.

**TABLE 1:** Computed gas‐phase complexation energies ∆*G*_complex_ of Cu^+^ and Ag^+^ with n C_2_H_2_ (in kJ mol^−1^) at the RI‐r^2^SCAN‐3c(D4)/def2‐mTZVPP, RIJCOSX‐B3LYP(D4)/def2‐TZVPP and DLPNO‐CCSD(T)/def2‐QZVPP//RI‐r^2^SCAN‐3c(D4)/def2‐mTZVPP levels of theory. For both [M(C_2_H_2_)_n−1_]^+^ and [M(C_2_H_2_)_n_]^+^, ground state structures were used for the calculations.

${\left[M{\left(C_{2}H_{2}\right)}_{n-1}\right]}^{+}+C_{2}H_{2}\overset{\Delta G_{\mathrm{complex}}}{\to }{\left[M{\left(C_{2}H_{2}\right)}_{n}\right]}^{+}$						
	M═Cu			M═Ag		
*n*	r^2^SCAN‐3c	B3LYP	DLPNO‐CCSD(T)	r^2^SCAN‐3c	B3LYP	DLPNO‐CCSD(T)
1	−198.9	−166.6	−136.5	−136.5	−121.7	−104.2
2	−166.0	−135.4	−130.0	−115.5	−96.7	−88.6
3	−49.2	−29.9	−25.6	−27.3	−18.5	−14.9
4	+4.2	+6.8	+7.2	−6.7	−2.4	−7.3

To further analyse the bonding situation in **1**, we performed EDA‐NOCV calculations (Figure 3 and section S5) [32]. The plots of the deformation densities Δ*ρ* for the two main orbital interactions Δ*E*_Orb(1)_ and Δ*E*_Orb(2)_ in [Cu(C_2_H_2_)_3_]^+^ (Figure 3A) show that the bonding can be described by mainly *σ*‐donation (51 %) and *π*‐backdonation (28 %), confirming the applicability of the Dewar‐Chatt‐Duncanson model to our system.

**FIGURE 3:**
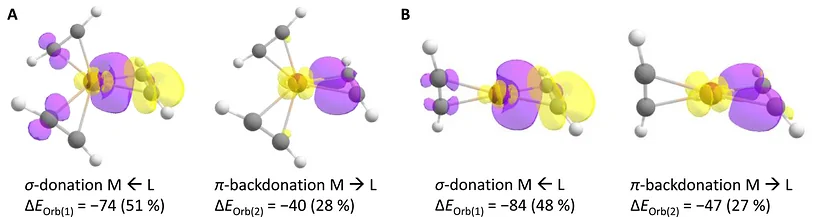
Plot of the deformation densities Δρ for the interactions between the fragments A) [Cu(C_2_H_2_)_2_]^+^ + C_2_H_2_ and B) [Cu(C_2_H_2_)]^+^ + C_2_H_2_ (Details: Figure S10 and Table S8). In both cases, the first and second contribution to the orbital interaction Δ*E*_Orb_ are shown. They represent σ‐donation and π‐backdonation. Energies given in kJ mol^−1^. Deformation densities plotted with an isovalue of 0.002, charge flows from yellow to purple.

We were further interested in the EDA‐NOCV analysis of hypothetical [Cu(C_2_H_2_)_2_]^+^, as our thermodynamic calculations indicated a pronounced decrease in bonding energy between the second and third coordinated acetylene ligand. While the bonding interaction (*σ*‐donation and *π*‐backdonation, Figure 3B) is very similar to [Cu(C_2_H_2_)_3_]^+^, the difference in bonding energy is less pronounced in the EDA‐NOCV results than in the complexation energies reported in Table 1. We attribute this discrepancy primarily to the different complex geometries used for [Cu(C_2_H_2_)_2_]^+^: the EDA‐NOCV analysis employs a bent structure as the [Cu(C_2_H_2_)_2_]^+^ fragment (43 kJ mol^−1^ higher in energy), whereas the complexation energies in Table 1 are based on the minimum structure of [Cu(C_2_H_2_)_2_]^+^ (see Figure S10 for the complex geometries and relative energies). For both complexes, [Cu(C_2_H_2_)_3_]^+^ and [Cu(C_2_H_2_)_2_]^+^, *σ*‐donation from the ligand HOMO into an empty Cu orbital was identified as the main bonding interaction. Since Cu(I) is a cationic closed shell *d* ^10^ system and hence expected to only slightly donate electron density back into the ligand LUMO, this contribution is only half that of *σ*‐donation, but it is still existing for **1**. The overall relatively small Cu(*η* [2]–C≡C) bonding interactions account for the observed only small red shift of the C≡C stretching vibration from 1974 (free HCCH) to 1900 cm^−1^ (**1**). This also becomes apparent in comparison to known systems with electron richer ligand environments as shown in Figure 1, that may red shift down to below 1800 cm^−1^ [5, 11].

## 3 Conclusion

The homoleptic acetylene complex [Cu(C_2_H_2_)_3_][Al(OR^F^)_4_] (**1**) has been synthesized using the weakly coordinated precursor [(N_2_)Cu{Al(OR^F^)_4_}]. NMR and Raman spectroscopic data, as well as quantum chemical calculations indicate stronger acetylene coordination in **1** compared to the silver analogue [Ag(C_2_H_2_)_3_][Al(OR^F^)_4_]. Furthermore, **1** adopts a *D*_3_‐symmetry of the cation (and not *C*_2_ as in the silver complex). Despite its slightly higher thermodynamic stability, **1** remains labile towards rapid decomposition at room temperature into elemental copper and polyacetylene, or copper acetylides. Therefore, it requires handling at low temperatures.

## Conflicts of Interest

All authors declare no conflicts of interest.

## Supporting information



The electronic supporting information contains the general synthetic methods and characterization techniques used for this work together with the experimental procedures. Additional figures such as NMR and Raman spectra are presented, as well as the crystallographic data of the isolated salt and details of the quantum chemical calculations. The authors have cited additional references within the supporting information.
**Supporting File 1**: chem71597‐sup‐0001‐SuppMat.pdf.

## Data Availability

X‐ray crystallographic data are available free of charge from the Cambridge Crystallographic Data Centre (CCDC) under the reference number 2566048 via https://www.ccdc.cam.ac.uk/structures/. All other data that support the findings of this study are contained in the main text, in the electronic supporting information or are available from the corresponding author upon reasonable request.
