# Supplementary material for: A Homoleptic Copper(I) Acetylene Complex
Source: Chemistry. 2026 Aug 21;32(42):e71597. doi: 10.1002/chem.71597 (PMC13755141; doi:10.1002/chem.71597)
Supplement: Supplementary file 1 [file CHEM-32-e71597-s001.pdf]

Supporting Information

A Homoleptic Copper(I) Acetylene Complex

Julie Willrett[a], Elisa Zöllner[a], Burkhard Butschke[a] and Ingo Krossing*[a]

[a] M. Sc. J. Willrett, B. Sc. Elisa Zöllner, Dr. Burkhard Butschke, Prof. Dr. I. Krossing, Institut für Anorganische und Analytische Chemie and Freiburger Materialforschungszentrum (FMF), Albert-Ludwigs-Universität Freiburg, Albertstr. 21, 79104 Freiburg, Germany.

* E-Mail: krossing@uni-freiburg.de

Table of contents

1. Abbreviations 2
2. Material and Methods 3
3. Detailed Synthesis of $[Cu(C_2H_2)_3][Al(OR^F)_4]$ (**1**) with NMR and Raman Spectra 5
4. Single-Crystal XRD Data 10
5. Computational details 22
6. References 27

1. Abbreviations

$[Al(OR^F)]^-$	= $[Al\{OC(CF_3)_3\}_4]^-$
DFT	= density functional theory
FTIR	= Fourier-transform infrared spectroscopy
FWHM	= full width at half maximum
OR^F	= $OC(CF_3)_3$
XRD	= X-ray diffraction

2. Material and Methods

General procedure

All manipulations were carried out under an argon or dinitrogen atmosphere and under exclusion of air and moisture using an MBraun glovebox filled with dinitrogen (O_2/H_2O < 1 ppm) and using standard Schlenk techniques. All reactions were carried out in Schlenk tubes with grease-free PTFE valves. All glassware was dried over-night in an oven at 150 °C and was thoroughly flame-dried prior to usage. The solvent pentafluorobenzene (5FB, ABCR) was pre-dried over CaH_2 and distilled onto and stored over activated 3 Å molecular sieves. *N*-pentane (VWR) was dried using a Grubbs apparatus and stored over 3 Å molecular sieves. Oxygen was removed from all solvents by three freeze-pump-thaw cycles. C_2H_2 (2.6, SWF) was used as received. $[(N_2)Cu\{Al(OR^F)_4\}]$ was prepared using a literature known synthesis.[1]

Vibrational Spectroscopy

Raman measurements were carried out at on a Vertex 70 IR spectrometer with installed RAM II Raman module (1064 nm exciting line of a Nd-YAG laser) and liquid nitrogen cooled Ge detector. Samples were measured in 5 mm NMR tubes with J. Young PTFE valve. Spectra were recorded at RT in a range from 4000-80 cm^{-1} and a resolution of 4 cm^{-1} with a laser power of 100 mW and 1,000 scans.
Spectroscopic data were processed with the Bruker OPUS 7.5 software and baseline corrections were carried out with five iterations. The signal intensity was normalized and the relative IR intensities were reported as followed with regard to the most intensive signal: ≥ 0.7 = very strong (vs), ≥ 0.6 strong (s), ≥ 0.5 = medium strong (ms), ≥ 0.4 = medium (m), ≥ 0.3 = medium weak (mw), ≥ 0.2 = weak (w), < 0.2 = very weak (vw). Raman intensities are not determined and reported due to considerable fluorescence.

NMR Spectroscopy

NMR spectra were recorded at RT on a Bruker Avance III HD 300 MHz. The samples were dissolved in 5FB (0.6 mL) in a 5 mm NMR tube with J. Young PTFE valve under inert conditions (argon atmosphere). The spectra were calibrated by using the ^{1}H signal of the solvent 5FB (δ = 7.016 ppm, rel. to TMS). The calibration of the spectra of other nuclei were adjusted accordingly using die IUPAC Ξ-table.[2] Spectroscopic data was analysed and plotted using Bruker TopSpin 4.1.3 software. ^{1}H NMR and ^{13}C NMR spectra are referenced against TMS, ^{19}F NMR spectra against $CFCl_3$, ^{27}Al NMR spectra against an aqueous solution of $Al(NO_3)_3$ (1.1 mM) and ^{63}Cu NMR spectra against $[Cu(CH_3CN)_4][ClO_4]$ + 10 % C_6D_6. The broad signal at around 60 ppm in the ^{27}Al spectra stems from the probe head.

Single Crystal X-Ray Diffraction

Single crystal X-ray diffraction data were acquired using a Bruker D8 VENTURE dual wavelength Mo/Cu three-circle diffractometer with a microfocus sealed X-ray tube using a mirror optics as monochromator and a Bruker PHOTON III detector. Crystal selection was performed at −40 °C under PFPE oil AB128330 (ABCR). Crystals were mounted on 0.1 to 0.2 mm diameter MiTeGen micromounts and were shock-cooled (unless stated otherwise) using an Oxford Cryostream 800 low temperature device.[3] Data were collected at 100(2) K, 150(2) K or 200(2) K using monochromatic MoK_α radiation (λ = 0.71073 Å). Crystallographic data were integrated with SAINT (version 8.40B)[4] and a multi-scan absorption correction using SADABS[5] was performed. Structures were solved by direct methods with SHELXT[6] and refined by full-matrix least-squares methods against F^2 by SHELXL-2019/3[7] using the GUI software ShelXle.[8] Refinement of disordered moieties was done using bond lengths restraints and displacement parameter restraints and was performed using the program DSR.[9,10] Finalization of the

gathered data was done with FinalCif.[11] Graphical representations of the crystal structures were created using Mercury (version 2022.3.0). Crystallographic data for the structures reported here have been deposited with the Cambridge Crystallographic Data Centre (CCDC number: 2566048).[12]

Computational Details

All quantum chemical calculations were performed with the ORCA programme package (version 5.0.3 or 6.1.1 for EDA-NOCV).[13,14] Density functional theory (DFT) was used either with the r^{2}SCAN-3c functional[15] and def2-mTZVPP basis set[16,17] with the resolution-of-identity (RI) approximation[18,19] and dispersion correction (D4),[20] or with the B3LYP functional[21–23] and def2-TZVPP basis set[16] with the resolution-of-identity (RI) approximation[17–19,24–26] and dispersion correction (D4).[20,27] All computed minimum structures were checked for the absence of imaginary vibrational frequencies.
Single point calculations were performed on the r^{2}SCAN-3c or B3LYP structures (as indicated) using the DLPNO-CCSD(T) method[28,29] with the def2-QZVPP basis set,[30] tight convergence criteria (tightscf) and normal DLPNO thresholds.
EDA-NOCV calculations[31] were performed with the ORCA programme package (version 6.1.1) at the RIJCOSX-B3LYP(D4)/def2-TZVPP level of theory. Plots of deformation densities $\Delta\rho$ were plotted with a grid size of 100 and isovalue of 0.002.
Raman activities were simulated at the RI-r^{2}SCAN-3c(D4)/def2-mTZVPP and the RIJCOSX-B3LYP(D4)/def2-TZVPP levels of theory and plotted with a FWHM of 30 cm^{-1}.
NMR shifts were simulated at the RIJCOSX-B3LYP(D4)/def2-TZVPP level of theory.

3. Detailed Synthesis of $[Cu(C_2H_2)_3][Al(OR^F)_4]$ (**1**) with NMR and Raman Spectra

$[(N_2)Cu\{Al(OR^F)_4\}]$ (15 mg, 0.014 mmol) was dissolved in 5FB (1.5 mL). The solution was frozen, evacuated, and then brought to room temperature to obtain the reaction mixture under the vapour pressure of the solvent. C_2H_2 gas (1 atmosphere) was added using an open system with a bubbler to avoid higher acetylene pressures. The mixture was stirred at −40 °C for 10 min and was then layered at −40 °C with *n*-pentane (8 mL). The mixture was stored in a freezer at −40 °C yielding colourless crystals of **1**. The crystallization appears to be quantitative, yet the product is not stable upon evacuation, so no yield was determined.

NMR spectra were measured under C_2H_2 atmosphere (1 bar). Raman spectra were measured of crystals of **1** under argon atmosphere.

^{1}H NMR (300.18 MHz, 5FB under 1 atm. C_2H_2, RT): δ = 3.28 (s, $[Cu(C_2\mathbf{H}_2)_3][Al(OR^F)_4]$, coalescence signal with C_2H_2 atmosphere) ppm. *Solvent signal visible at 7.02 ppm.*

^{13}C{^{1}H} NMR (75.48 MHz, 5FB under 1 atm. C_2H_2, RT): δ = 72.9 (s, $[Cu(\mathbf{C}_2H_2)_3][Al(OR^F)_4]$, coalescence signal with C_2H_2 atmosphere) ppm. *Solvent signals visible at 148.3-144.5,143.7-139.8, 139.5-135.6 and 100.3-99.5 ppm. Anion signals not visible.*

^{19}F NMR (376.53 MHz, 5FB under 1 atm. C_2H_2, RT): δ = −76.4 (s, $[Cu(C_2H_2)_3][Al(OC(C\mathbf{F}_3)_3)_4]$) ppm. *Solvent signals visible at −141.2, −156.9 and −165.2 ppm.*

^{27}Al NMR (78.22 MHz, 5FB under 1 atm. C_2H_2, RT): δ = 34.5 (s, $[Cu(C_2H_2)_3][\mathbf{Al}(OR^F)_4]$) ppm. *Anion decomposition product visible at 46.6 ppm.*

^{63}Cu NMR (79.59 MHz, 5FB under 1 atm. C_2H_2, RT): δ = −2425 (s, $[\mathbf{Cu}(C_2H_2)_3][Al(OR^F)_4]$) ppm.

Raman spectrum of crystals of **1**, embedded in a solvent mixture of 5FB and n-pentane. A reference spectrum for the assignment of solvent bands is included with Figure S 6. Full assignment in Table S1.

FT Raman (1,000 scans, 100 mW): $\tilde{\nu}$/cm^{-1} = 3097 (vw), 2964 (w), 2937 (w), 2906 (w), 2877 (m), 2727 (vw), 1900 (m), 1648 (vw), 1463 (vw), 1440 (vw), 1410 (vw), 1305 (vw), 1284 (vw), 1139 (vw), 1073 (vw), 1030 (vw), 867 (vw), 840 (vw), 798 (w), 746 (w), 719 (s), 578 (vs), 538 (w), 470 (m), 435 (m), 392 (m), 366 (m), 325 (s), 288 (m), 233 (m).

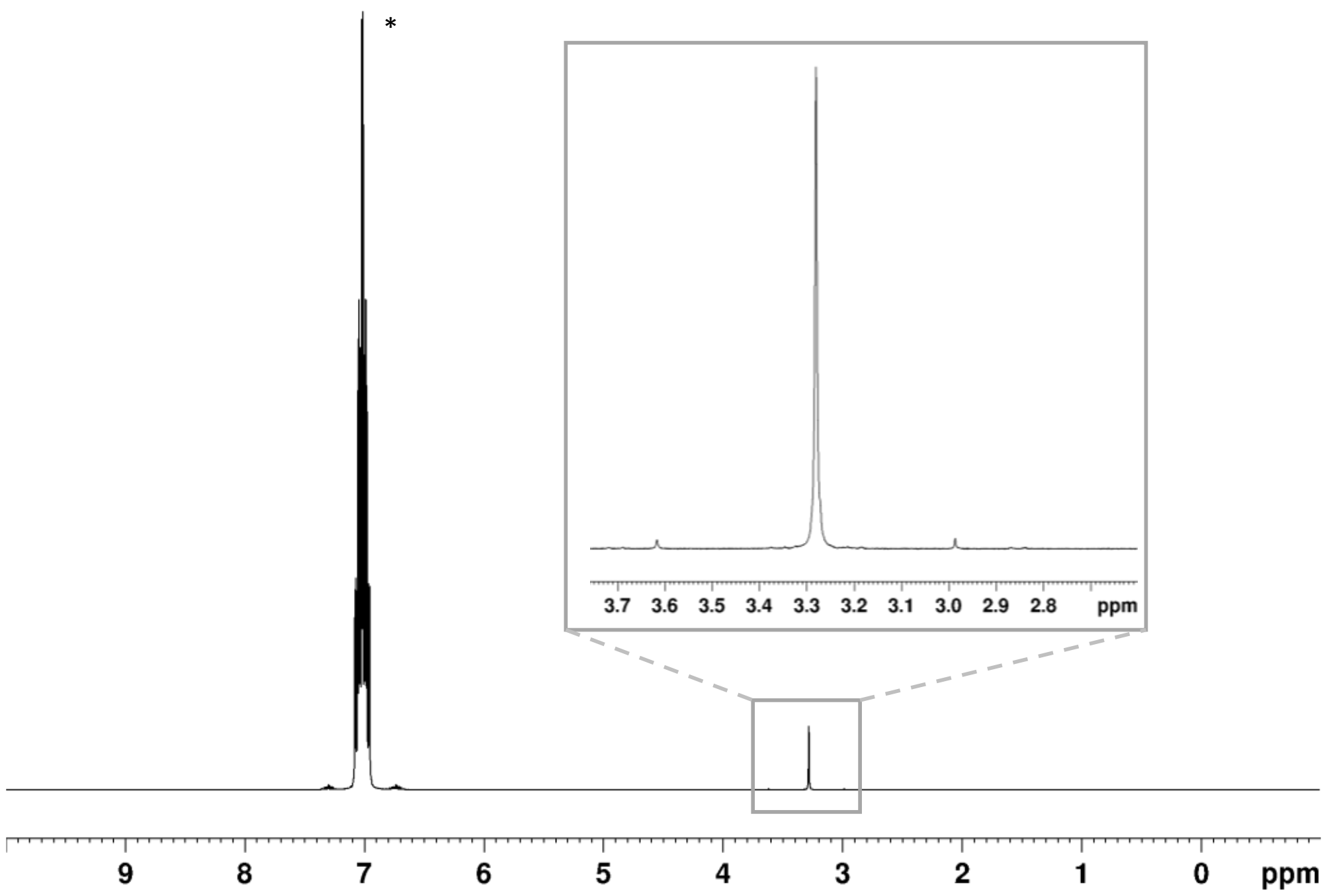

Figure S 1: ^{1}H NMR spectrum (300.18 MHz, 5FB under 1 atm. C_2H_2, RT) of $[Cu(C_2H_2)_3][Al(OR^F)_4]$ **1**. Signals stemming from the solvent 5FB marked with *.

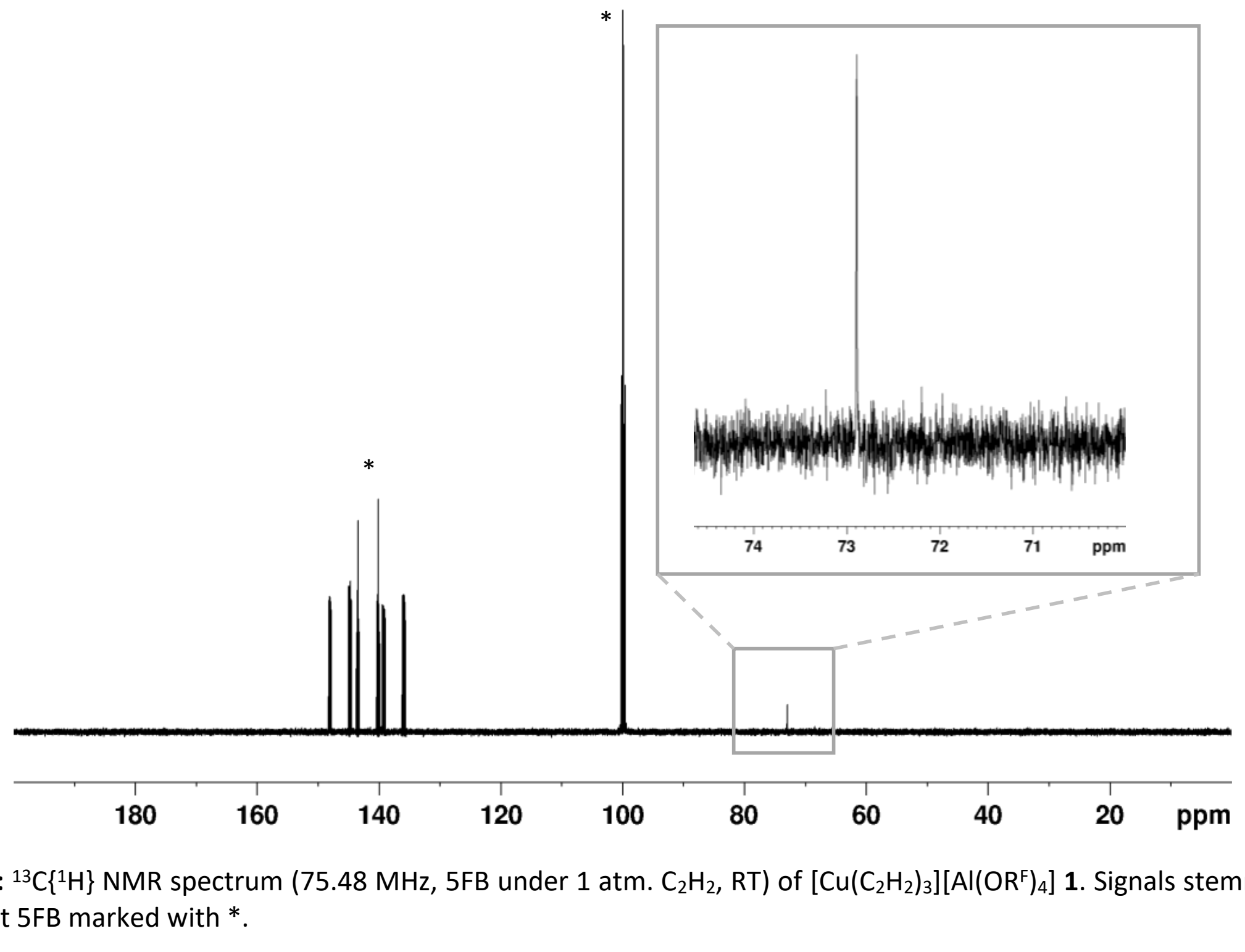

Figure S 2: $^{13}C\{^{1}H\}$ NMR spectrum (75.48 MHz, 5FB under 1 atm. C_2H_2, RT) of $[Cu(C_2H_2)_3][Al(OR^F)_4]$ **1**. Signals stemming from the solvent 5FB marked with *.

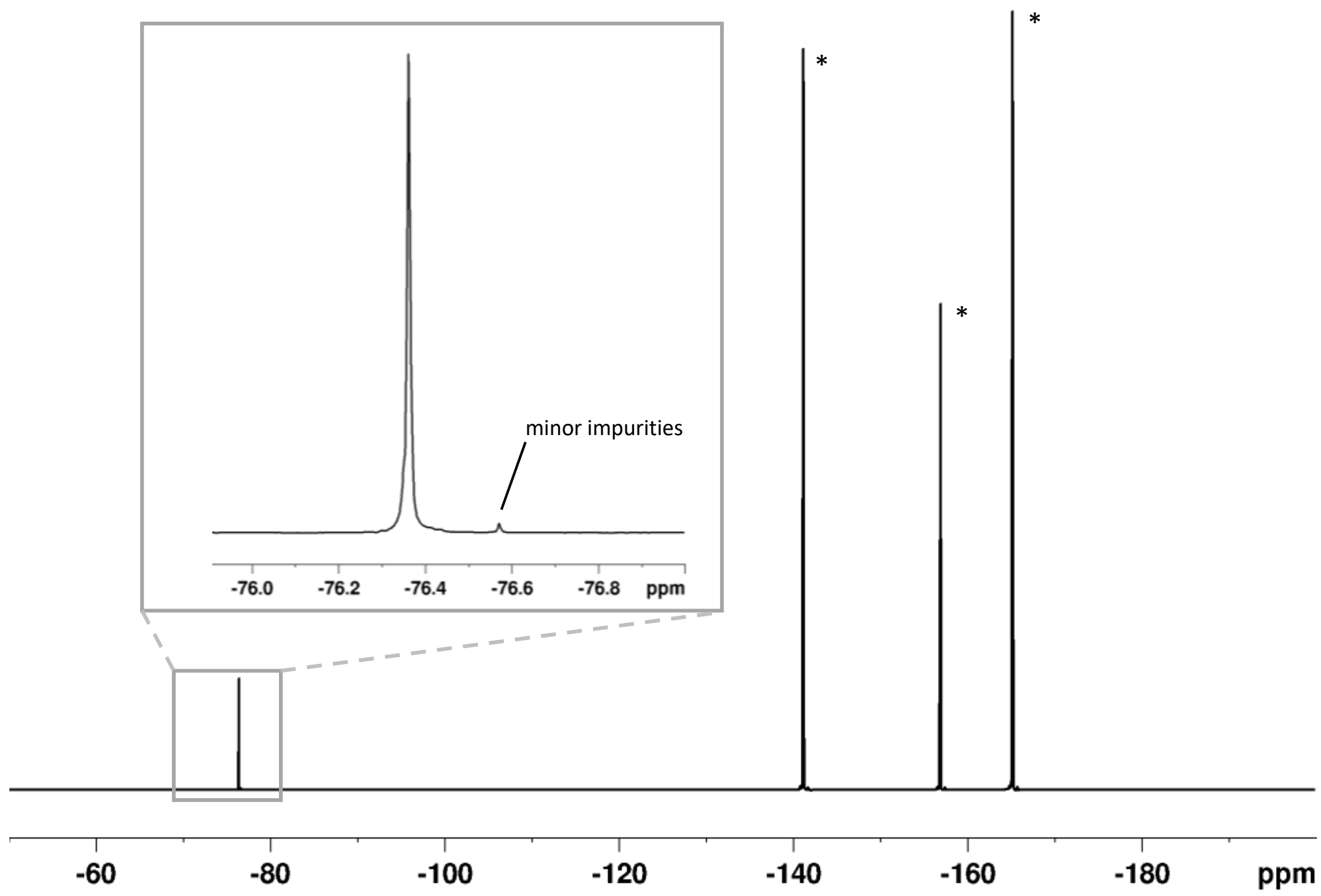

Figure S 3: ^{19}F NMR spectrum (376.53 MHz, 5FB under 1 atm. C_2H_2, RT) of $[Cu(C_2H_2)_3][Al(OR^F)_4]$ **1**. Signals stemming from the solvent 5FB marked with *.

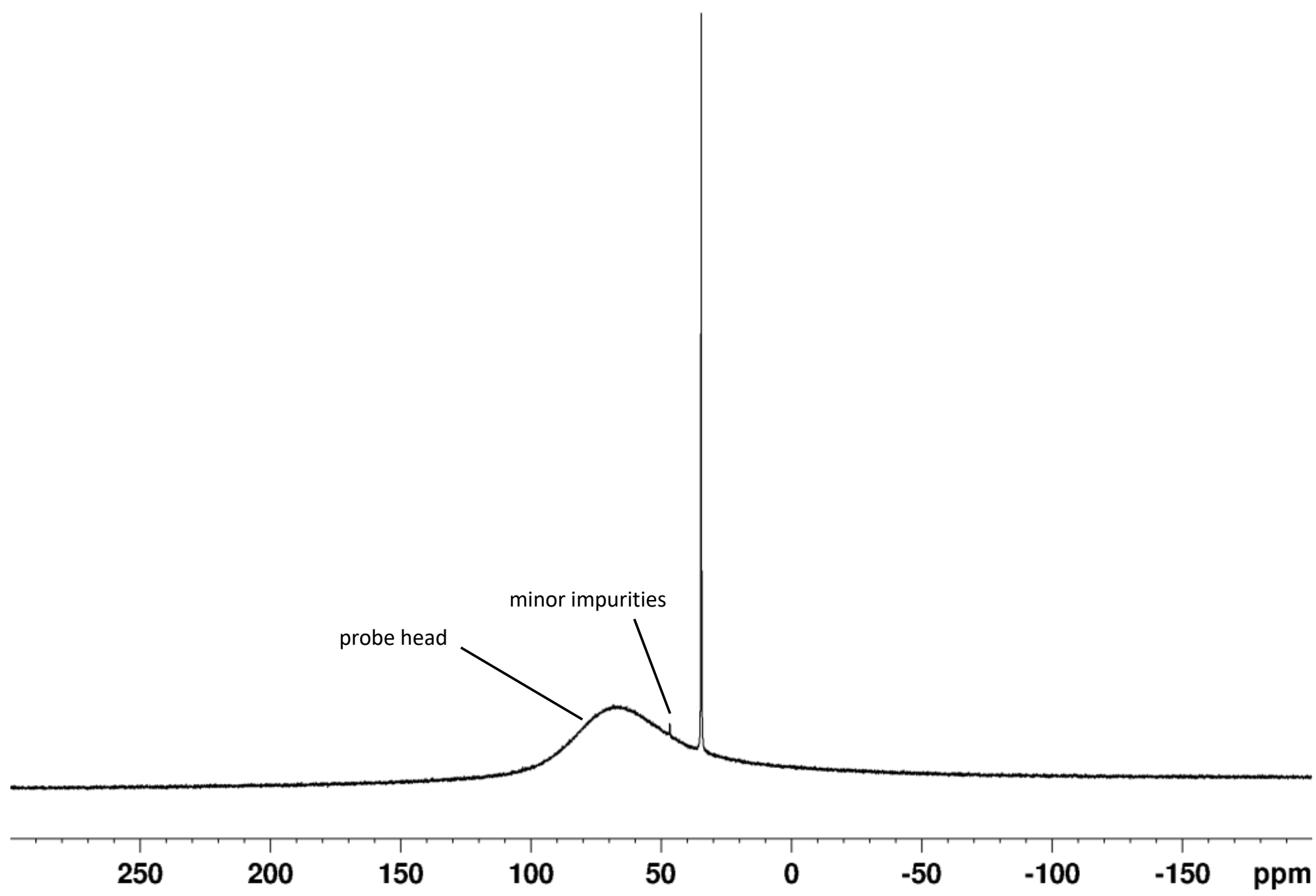

Figure S 4: ^{27}Al NMR spectrum (78.22 MHz, 5FB under 1 atm. C_2H_2, RT) of $[Cu(C_2H_2)_3][Al(OR^F)_4]$ **1**.

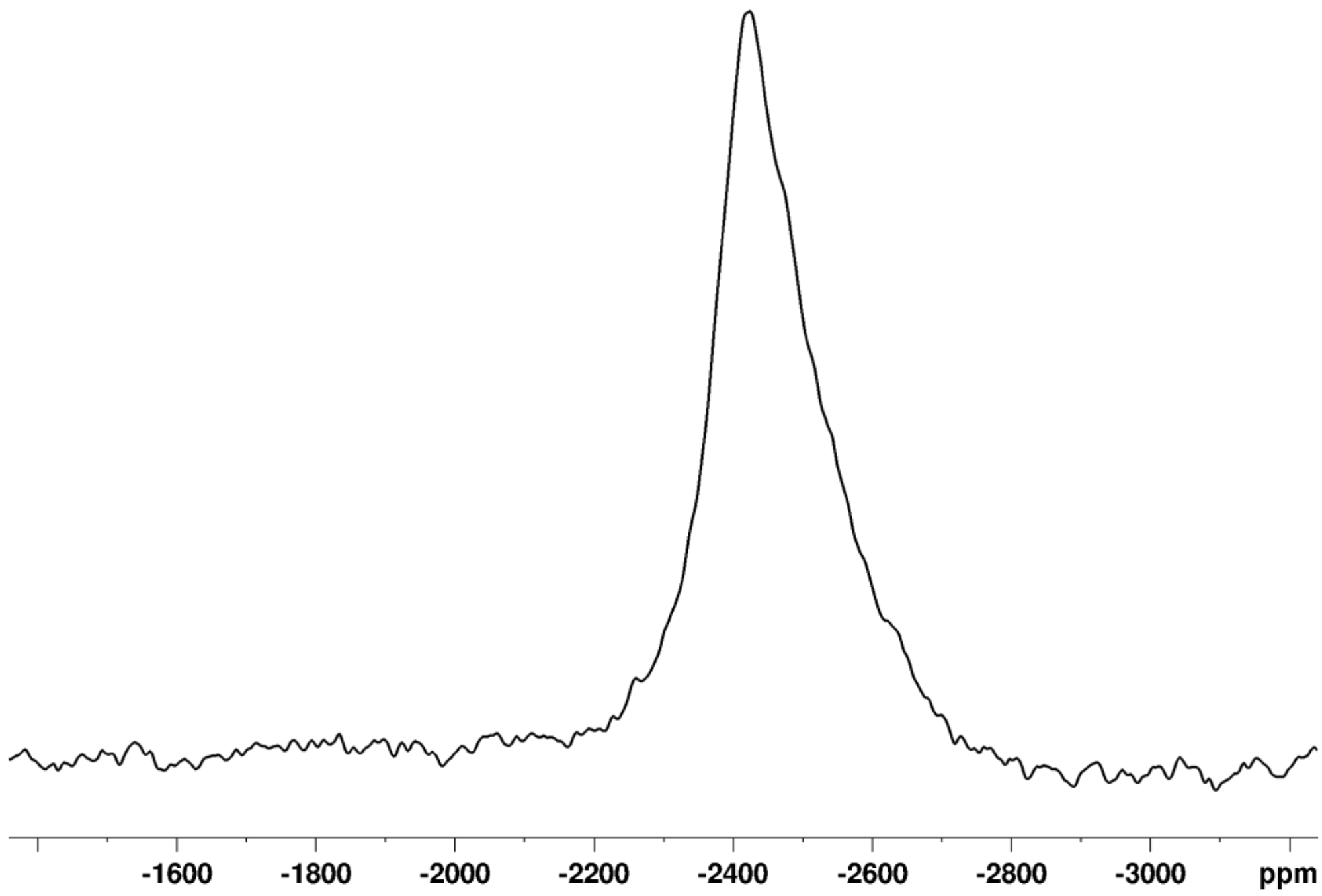

Figure S 5: ^{63}Cu NMR spectrum (79.59 MHz, 5FB under 1 atm. C_2H_2, RT) of $[Cu(C_2H_2)_3][Al(OR^F)_4]$ **1**.

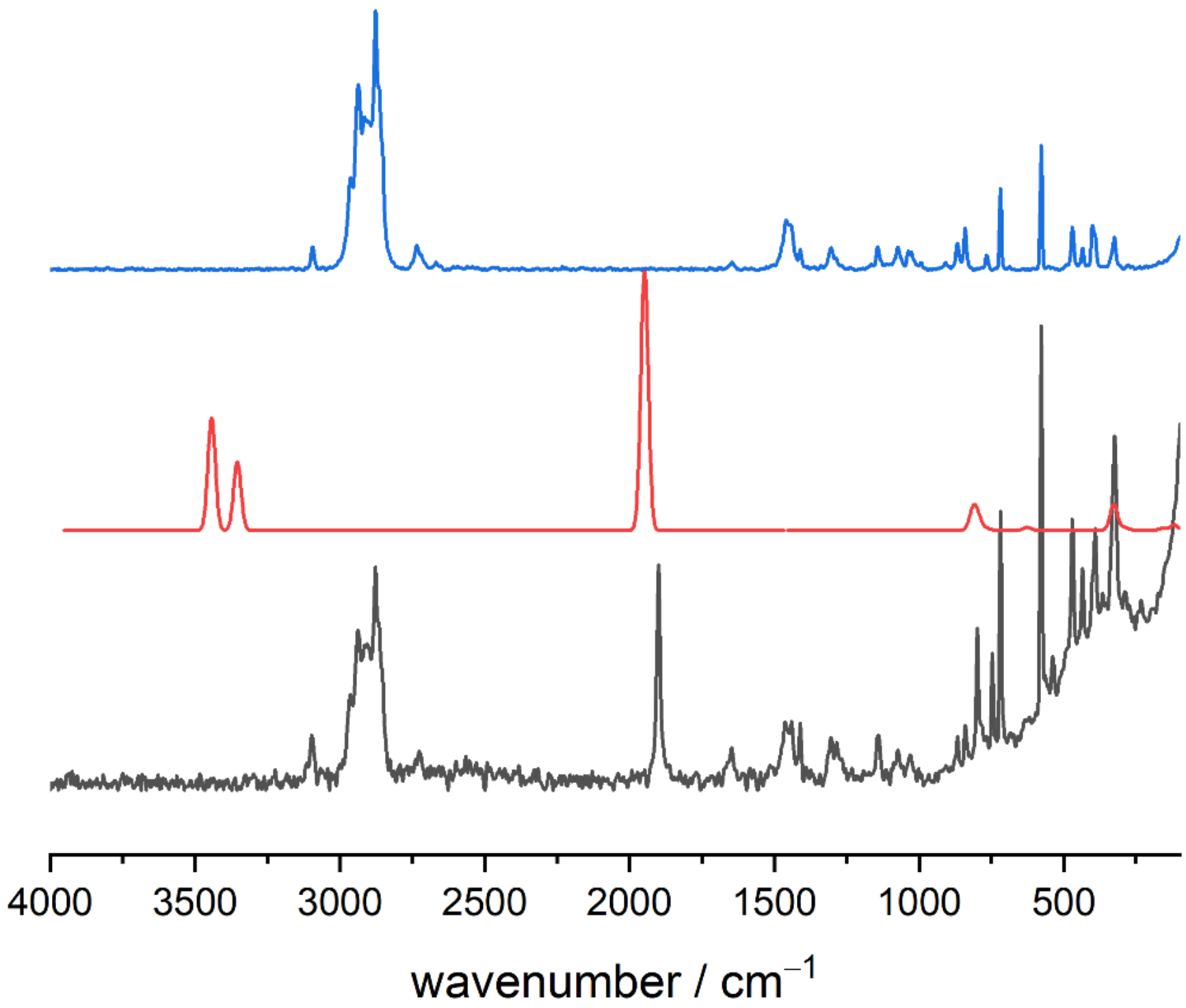

Figure S 6: Experimental Raman spectrum of crystals of $[Cu(C_2H_2)_3][Al(OR^F)_4]$ **1** (black, 1,000 scans, 100 mW), calculated Raman spectrum of $[Cu(C_2H_2)_3]^+$ (red, r2SCAN-3c/def2-mTZVPP level of DFT) and experimental Raman spectrum of a mixture (1:5) of 5FB and *n*-pentane (blue, 1,000 scans, 100 mW).

Table S 1: Assignment of IR and Raman vibrations of $[Cu(C_2H_2)_3][Al(OR^F)_4]$ (**1**) and comparison with the calculated spectra for cation bands (r^2SCAN-3c/def2-mTZVPP and B3LYP/def2-TZVPP levels of DFT), with the reference compound $[NEt_4][Al(OR^F)_4]$ for anion bands,[32] and with the reference spectrum of a mixture of 5FB and *n*-pentane (1:5). Intensities for bands in the experimental spectrum of **1** < 800 cm^{-1} deviate strongly from the references due to the uneven baseline of the spectrum and in general due to the normalizations of the separate spectra. Anion bands marked in grey.

**1** exp. Raman	**1⁺** calc. Raman r^2SCAN/B3LYP	$[Al(OR^F)_4]^-$ exp. Raman	**5FB / *n*-pentane** exp. Raman	**Assignment** a)
233 (m)		234 (w)		–
288 (m)		289 (w)		C–C
325 (s)	328 (vw)/ 294 (vw)	323 (ms)	326 (vw)	C–C, Al–O
366 (m)		368 (w)		C–C, C–F, Al–O
392 (m)			401 (vw)	–
435 (m)			435 (vw)	–
470 (m)			470 (vw)	–
538 (w)		538 (w)		C–C, C–O
–		563 (w)		C–C, Al–O
578 (vs)			578 (m)	–
719 (s)			719 (w)	–
746 (w)		747 (ms)		–
–			766 (vw)	–
798 (w)	797 (vw)/ 801 (vw)	798 (s)		–
840 (vw)		834 (w)	840 (vw)	C–C, Al–O
867 (vw)			868 (vw)	–
–			909 (vw)	–
–			993 (vw)	–
–		978 (mw)		C–C, C–F
1030 (vw)			1037 (vw)	–
1073 (vw)			1073 (vw)	–
1139 (vw)		1139 (mw)		C–C, C–F
–		1173 (mw)		C–C, C–F
–		1235 (mw)		C–C, C–F
1284 (vw)		1274 (mw)		C–C, C–F
1305 (vw)		1300 (m)	1304 (vw)	C–C, C–F
1410 (vw)			1410 (vw)	–
1440 (vw)				
1463 (vw)			1459 (w)	–
1648 (vw)			1645 (vw)	–
1900 (m)	1948 (vs)/ 1979 (vs)			acetylene C–C
2727 (vw)			2734 (vw)	–
2877 (m)			2877 (vs)	–
2906 (w)			2915 (m)	–
2937 (w)			2936 (s)	–
2964 (w)			2963 (w)	–
3097 (vw)			3095 (vw)	–
–	3354 (w)/ 3357 (w)			acetylene C–H
–	3441 (m)/ 3448 (m)			acetylene C–H

a) From a visualization of the calculated spectra and from reference [32].

4. Single-Crystal XRD Data

Table S 2: Crystallographic data of $[Cu(C_2H_2)_3][Al(OR^F)_4]$ **1**. **1** was refined as a 2-component inversion twin.

	**1**
**CCDC number**	2566048
**Empirical formula**	$C_{22}H_6AlCuF_{36}O_4$
**Formula weight**	1108.79
**Temperature [K]**	100(2)
**Crystal system**	monoclinic
**Space group (number)**	$P2_1$ (4)
a **[Å]**	9.491(5)
b **[Å]**	15.240(9)
c **[Å]**	12.258(6)
**α [°]**	90
**β [°]**	100.68(2)
**γ [°]**	90
**Volume [Å^3]**	1742.3(16)
Z	2
ρ_{calc} **[g cm^{-3}]**	2.113
μ **[mm^{-1}]**	0.878
***F*(000)**	1072
**Crystal size [mm^3]**	0.147×0.226×0.320
**Crystal colour**	colourless
**Crystal shape**	block
**Radiation**	MoK_α (λ=0.71073 Å)
**2θ range [°]**	3.38 to 51.77 (0.81 Å)
**Index ranges**	−11 ≤ h ≤ 11<br>−18 ≤ k ≤ 18<br>−15 ≤ l ≤ 15
**Reflections collected**	38411
**Independent reflections**	6722<br>R_{int} = 0.0737<br>R_{sigma} = 0.0477
**Completeness to θ = 25.242°**	99.9 %
**Data / Restraints / Parameters**	6722 / 11807 / 1186
**Absorption correction T_{min}/T_{max} (method)**	0.5844 / 0.7453 (multi-scan)
**Goodness-of-fit on F^2**	2.130
**Final *R* indexes [*I* ≥ 2σ(*I*)]**	R_1 = 0.1162<br>wR_2 = 0.2927
**Final *R* indexes [all data]**	R_1 = 0.1585<br>wR_2 = 0.3176
**Largest peak/hole [eÅ^{-3}]**	0.74/−0.96

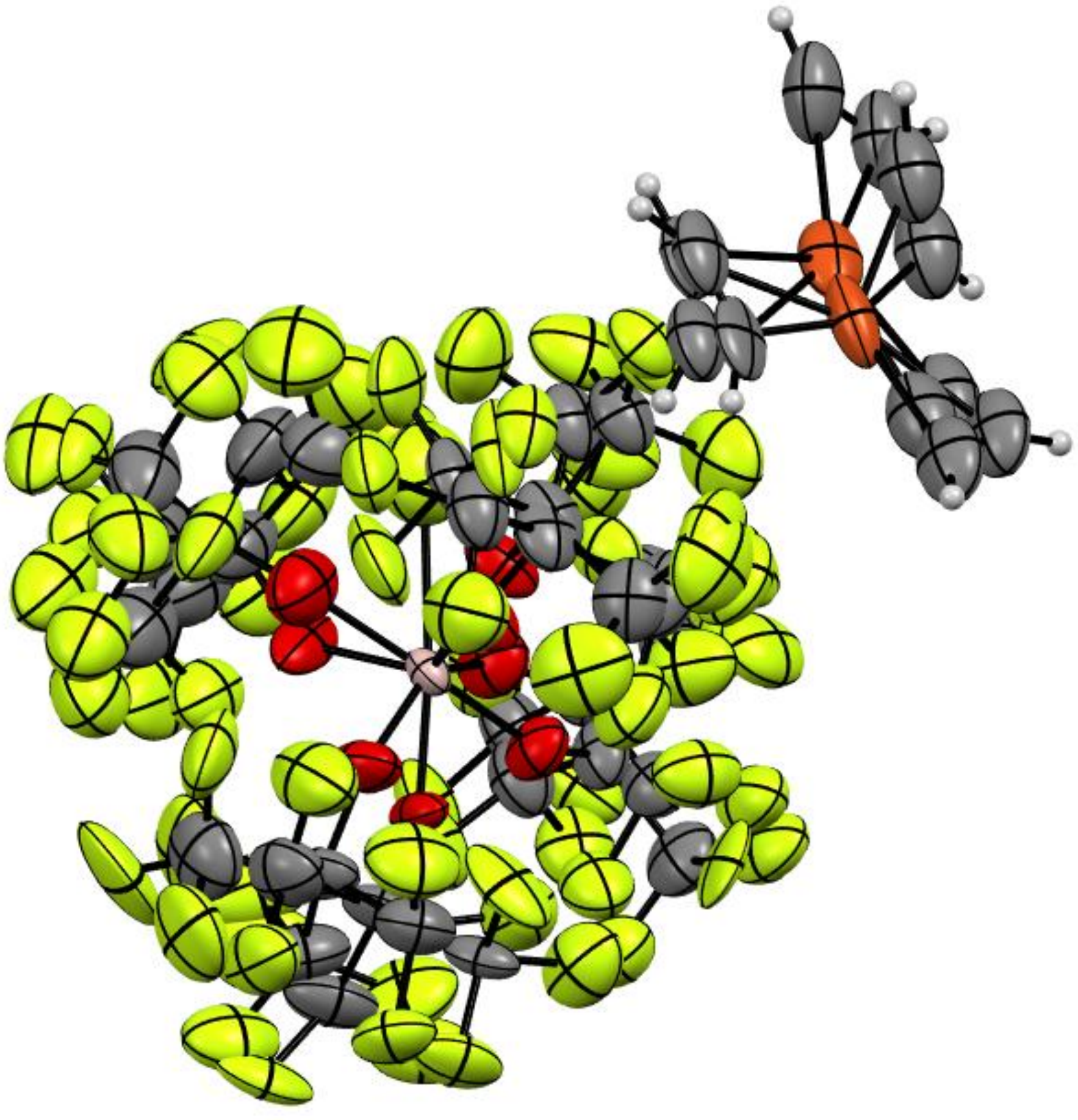

Figure S 7: Molecular structure of **1** with disorders in the anion and the cation shown.

Detailed Description of the Treatment of the Crystallographic Data and Discussion of the Inherent Crystallographic Challenges

The lattice of the reflections of a normal crystal can be described using the basic vectors **a***, **b***and **c***. Consequently, the positions of the Bragg reflections are given by

$$\mathbf{H} = h\mathbf{a}^* + k\mathbf{b}^* + l\mathbf{c}^*. \tag{S1}$$

This is true for ideal crystals with perfect lattice periodicity. However, in some crystals (and also in **1**), a long-range order can be observed, which deviates slightly from lattice periodicity. In these cases, we call the crystal structure 'modulated'. This modulation can be addressed by expanding Eq. S1 by introducing modulation vectors **q**:[33]

$$\mathbf{H} = h\mathbf{a}^* + k\mathbf{b}^* + l\mathbf{c}^* + m_1\mathbf{q}_1 + m_2\mathbf{q}_2 + \ldots + m_d\mathbf{q}_d. \tag{S2}$$

Each modulation vector can be written as:[33]

$$\mathbf{q} = \sigma_1\mathbf{a}^* + \sigma_2\mathbf{b}^* + \sigma_3\mathbf{c}^*, \tag{S3}$$

and describes additional, so-called satellite reflections, which are generally of weaker intensity than the main reflections, and which do not obey space group symmetry. We speak of an incommensurate modulation if at least one component σ of a modulation vector is an irrational number, and then we enter crystallographic superspace.[34] Here, the 230 crystallographic space groups are not applicable anymore, and we would need superspace groups to adequately describe the structure.[33,34]

The two main origins of a modulated structure are displacive and occupational modulations,[33] of which the former one is most probably the reason for our crystallographic problems: Here, atomic positions differ from the basic structure. We suspect our large anion $[Al(OR^F)_4]^-$ to be the reason why we frequently observe modulation in our structures. The crystal structure is mainly defined by the anion positions, leaving a lot of space and mobility for the usually smaller cations and making displacive modulation probable. Furthermore, the alkoxy residues of the anion can occupy two main distinct conformations, which often leads to disorders in the anion. If this disorder is not statistical anymore, but (quasi) periodic, this can also lead to (incommensurate) modulation.

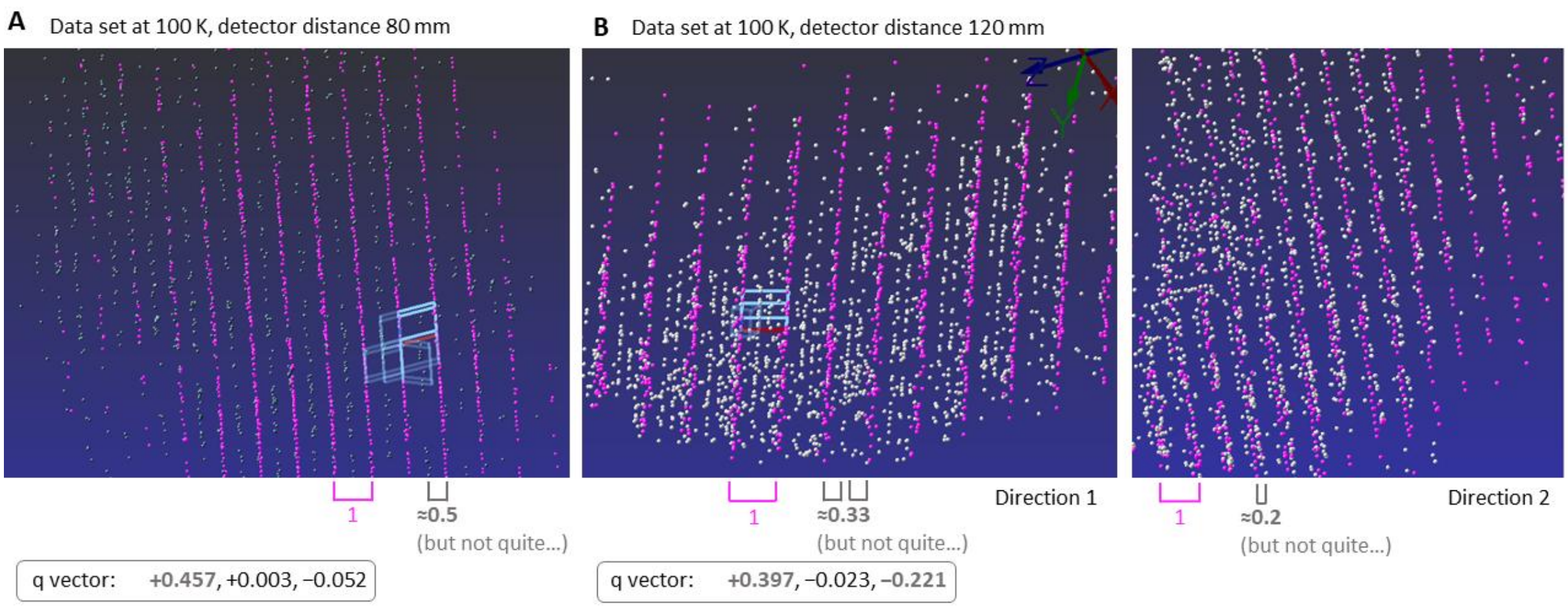

Figure S 8: Representations of the reciprocal space of two different crystals of **1**. Reflections belonging to the unmodulated part of the structure coloured in pink, satellite reflections coloured in grey. A) Reflections of the first crystal, measured with a detector distance of 80 mm. Satellites appear to be approximately in the middle of solid rows of unmodulated reflections. B) Reflections of the second crystal, measured with a detector distance of 120 mm, allowing for a better separation of satellites. The modulation becomes visible in two directions and hints at an incommensurately modulated structure.

In Figure S 7, images of the reciprocal space for **1** are shown, where the satellite reflections (in grey) are clearly visible as weaker lines between the more solid lines of the main lattice (in pink). At smaller detector distances, the satellites appear to be half-way in between reflections of the main lattice. At this point, it seemed still possible that our structure could be commensurately modulated and that we could address that by doubling the unit cell. Yet, by looking at the modulation vector, we saw that the first component is not 0.5 but 0.457. This could potentially be an artefact of the imprecise determination of the q vector, and we measured a different crystal again at a larger detector distance to reproduce the data or to potentially gather better data. Here, the full extent of the crystallographic difficulties became visible: The satellite reflections were now better separated and approximately at 1/3 of the main reflections (that would already mean tripling of the cell and would inevitably lead to bad data/parameter ratios…). Yet, they are only *approximately* at 1/3, the q vector can be determined with the component 0.397 (not 0.333333…). Furthermore, the satellites are visible in a second direction, reflected by the second nonzero component of the q vector of −0.221.

We then tried to measure the crystals at higher temperatures (200 K instead of 100 K), allowing more thermal motion, with the hope for the modulation to become a statistic disorder. Furthermore, we did not shock cool the crystals but applied a temperature ramp starting at −40 °C where we also picked the crystals. However, the modulation stayed the same and the only effect we could observe was an overall weaker diffraction of the crystal.

The incommensurate nature of the modulation is reflected by the ambiguity of the space group: The PLATON diagnostic program AddSym[35] proposes $P\overline{1}$, $P1$, Pm, $P2_1/c$ or $P2_1/m$ as space groups. This is rather unusual, as PLATON in most cases proposes only one space group. In our case, no solution in any of the proposed space groups leads to good results, probably because all these space groups are not applicable, and we would need a superspace group. However, this is not possible in standard crystallographic programs (and close to impossible in specialised programs such as JANA for complex molecular structures).

We tried to solve the structure in the triclinic and monoclinic crystal systems and with the single and double unit cell (one axis can be determined as 9.49 Å or 18.98 Å) and tried each permutation. The best results were obtained for the solution in monoclinic $P2_1$ (with implied inversion twinning) for the small cell. In this solution, we ignored the satellite reflections for the integration and treated the modulation as disorder. The modulation (and the fact that we ignored it) leads to high *R* values and general alerts. The fact that we used the small cell and ignored a large part of our data leads to a bad data/parameter ratio. However, we used restraints to describe the disorders, which could be regarded as additional data. In this description, the entire $[Cu(C_2H_2)_3]^+$ moiety is disordered, with the copper atom occupying two distinct sites with an occupation of approximately 0.53:0.47 (Figure S 9). This disorder is also visible in solutions in the larger cell, in which the free variable of the disorder is approximately 0.7-0.8. This shows that cell doubling does not solve the problem.

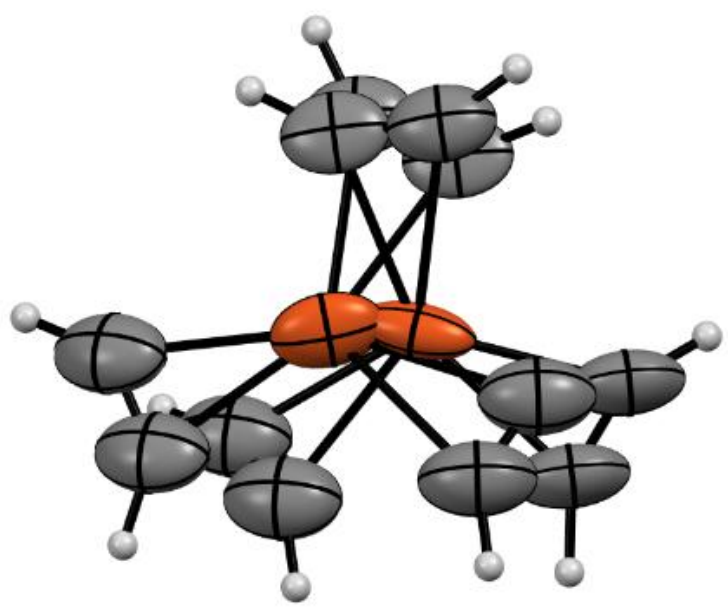

Figure S 9: Disorder of the cationic part of **1**.

To build a model of the disordered cations, we applied restraints (SADI, SIMU and RIGU). SADIs were applied on C−C bonds and Cu−C bonds. These are reasonable restraints, as we know by Raman spectroscopy of the crystals that all acetylene ligands must be equal as there is only one Raman band of the C≡C stretching vibration. The slightly cylindric ellipsoids in the cationic moiety indicate possible additional disorders, however, these do not considerably improve the model when implemented.

Acetylenic hydrogen atoms were refined freely and not using a riding model (DFIX with length of 0.95, SADI, FLAT on each individual $Cu-C_2H_2$ unit). Due to these treatments and the overall quality of the data, we do not discuss bond lengths and angles in the cation.

As a conclusion, we would like to emphasize that these results are highly reproducible. We could observe this modulation in each crystal that we measured, independent on the temperature. However, the connectivity of the structure becomes very clear despite the crystallographic problems and matches the NMR, Raman and computational data very well. We therefore chose to implement the structure in our manuscript and to discuss the issues with the structure transparently.

Atom Coordinates, Bond Lengths, Bond Angles

Table S 3: Atomic coordinates and U_{eq} [Å^2] for $[Cu(C_2H_2)_3][Al(OR^F)_4]$ **1**.

**Atom**	***x***	***y***	***z***	U_{eq}
Cu1	−0.1305(6)	0.4414(7)	0.2656(5)	0.071(2)
C1	0.049(3)	0.430(3)	0.187(3)	0.076(6)
H1	0.109(13)	0.382(9)	0.173(11)	0.091
C2	0.026(3)	0.499(3)	0.183(3)	0.071(6)
H2	0.04(2)	0.555(6)	0.154(16)	0.085
C3	−0.252(4)	0.558(2)	0.290(3)	0.091(6)
H3	−0.282(15)	0.606(7)	0.242(13)	0.109
C4	−0.233(4)	0.524(3)	0.367(3)	0.094(6)
H4	−0.26(2)	0.513(15)	0.438(9)	0.113
C5	−0.246(4)	0.338(2)	0.323(3)	0.085(6)
H5	−0.307(11)	0.337(13)	0.376(8)	0.102
C6	−0.187(5)	0.3044(18)	0.269(3)	0.087(6)
H6	−0.18(2)	0.254(9)	0.223(16)	0.104
Cu1A	−0.1283(8)	0.4996(8)	0.2706(6)	0.074(3)
C1A	0.005(4)	0.485(3)	0.149(3)	0.073(7)
H1A	0.051(12)	0.514(14)	0.097(8)	0.088
C2A	−0.003(4)	0.419(3)	0.182(3)	0.070(6)
H2A	0.03(2)	0.362(6)	0.166(17)	0.084
C3A	−0.167(5)	0.362(2)	0.302(4)	0.090(6)
H3A	−0.165(14)	0.305(7)	0.273(18)	0.107
C4A	−0.194(5)	0.398(3)	0.369(3)	0.090(6)
H4A	−0.22(2)	0.388(15)	0.439(9)	0.108
C5A	−0.228(5)	0.610(3)	0.329(4)	0.090(7)
H5A	−0.288(10)	0.627(15)	0.380(10)	0.108
C6A	−0.175(5)	0.6397(19)	0.268(4)	0.091(7)
H6A	−0.17(2)	0.696(7)	0.238(17)	0.109
Al1	0.5587(3)	0.4643(4)	0.7599(2)	0.0316(7)
O1_5	0.667(3)	0.5480(16)	0.721(2)	0.086(5)
C1_5	0.754(2)	0.6228(15)	0.7407(17)	0.085(4)
C2_5	0.663(3)	0.6954(14)	0.773(2)	0.091(5)
F1_5	0.660(3)	0.6865(17)	0.8819(18)	0.112(7)
F2_5	0.699(3)	0.7792(13)	0.7610(19)	0.100(7)
F3_5	0.530(3)	0.695(2)	0.713(3)	0.122(8)
C3_5	0.792(3)	0.6410(19)	0.627(2)	0.097(6)
F4_5	0.830(3)	0.5701(18)	0.575(2)	0.106(7)
F5_5	0.678(3)	0.6758(17)	0.559(2)	0.106(7)
F6_5	0.902(3)	0.697(2)	0.645(3)	0.113(9)
C4_5	0.886(3)	0.5998(18)	0.827(2)	0.101(5)
F7_5	0.961(3)	0.539(2)	0.783(3)	0.119(7)
F8_5	0.970(3)	0.668(2)	0.860(3)	0.120(9)
F9_5	0.843(3)	0.5638(16)	0.9145(18)	0.087(6)
O1_6	0.696(2)	0.5331(14)	0.796(2)	0.075(5)
C1_6	0.730(2)	0.6231(16)	0.7844(18)	0.090(5)
C2_6	0.819(3)	0.6118(18)	0.692(2)	0.103(5)
F1_6	0.742(3)	0.5645(19)	0.6085(19)	0.102(7)
F2_6	0.858(3)	0.688(2)	0.655(3)	0.098(8)
F3_6	0.933(3)	0.558(2)	0.717(2)	0.111(7)
C3_6	0.817(3)	0.6500(18)	0.895(2)	0.105(6)
F4_6	0.745(3)	0.669(2)	0.976(2)	0.126(8)
F5_6	0.904(3)	0.5864(17)	0.944(3)	0.113(8)
F6_6	0.906(3)	0.717(2)	0.883(3)	0.121(10)

C4_6	0.587(3)	0.667(2)	0.744(2)	0.110(6)
F7_6	0.516(3)	0.702(2)	0.817(2)	0.117(7)
F8_6	0.574(4)	0.715(3)	0.651(3)	0.145(10)
F9_6	0.479(3)	0.607(2)	0.709(3)	0.147(9)
O1_7	0.463(2)	0.4471(17)	0.6334(15)	0.071(5)
C1_7	0.343(2)	0.4605(14)	0.5504(17)	0.065(3)
C2_7	0.270(2)	0.5475(15)	0.5527(18)	0.068(4)
F1_7	0.352(3)	0.6120(19)	0.519(3)	0.126(8)
F2_7	0.147(3)	0.554(2)	0.4813(19)	0.072(6)
F3_7	0.254(3)	0.564(2)	0.6567(17)	0.114(7)
C3_7	0.236(3)	0.3880(17)	0.563(2)	0.085(5)
F4_7	0.319(3)	0.3228(17)	0.609(2)	0.109(7)
F5_7	0.155(3)	0.414(2)	0.635(2)	0.110(7)
F6_7	0.154(4)	0.362(3)	0.469(3)	0.107(10)
C4_7	0.400(2)	0.4512(17)	0.4433(18)	0.071(5)
F7_7	0.454(3)	0.3709(15)	0.445(2)	0.104(7)
F8_7	0.301(3)	0.471(2)	0.354(2)	0.074(7)
F9_7	0.508(2)	0.5060(19)	0.435(2)	0.099(7)
O1_8	0.419(2)	0.4909(16)	0.6560(15)	0.058(4)
C1_8	0.334(2)	0.4745(14)	0.5539(17)	0.062(4)
C2_8	0.416(2)	0.4917(17)	0.4609(18)	0.063(5)
F1_8	0.442(3)	0.5749(17)	0.438(3)	0.104(7)
F2_8	0.342(3)	0.463(3)	0.362(2)	0.073(7)
F3_8	0.5378(19)	0.448(2)	0.475(2)	0.104(8)
C3_8	0.271(3)	0.3816(15)	0.541(2)	0.076(5)
F4_8	0.243(4)	0.3520(18)	0.636(2)	0.100(7)
F5_8	0.350(4)	0.3202(18)	0.504(2)	0.110(7)
F6_8	0.143(4)	0.384(3)	0.469(3)	0.105(10)
C4_8	0.209(3)	0.5376(15)	0.546(2)	0.067(5)
F7_8	0.116(3)	0.507(2)	0.610(2)	0.103(7)
F8_8	0.144(3)	0.548(2)	0.4411(18)	0.066(6)
F9_8	0.236(3)	0.6177(14)	0.588(2)	0.102(7)
O1_9	0.493(2)	0.5025(14)	0.8691(16)	0.067(4)
C1_9	0.4135(18)	0.4944(11)	0.9525(15)	0.058(3)
C2_9	0.518(2)	0.5230(15)	1.0571(18)	0.073(5)
F1_9	0.5349(17)	0.6100(12)	1.0544(18)	0.077(5)
F2_9	0.474(2)	0.4972(17)	1.1478(15)	0.084(5)
F3_9	0.6494(18)	0.4857(16)	1.0680(19)	0.091(6)
C3_9	0.366(2)	0.4012(13)	0.9609(17)	0.063(4)
F4_9	0.303(2)	0.3615(13)	0.8698(16)	0.086(5)
F5_9	0.479(2)	0.3518(14)	1.0063(16)	0.081(5)
F6_9	0.267(3)	0.3932(16)	1.027(2)	0.088(6)
C4_9	0.284(2)	0.5564(14)	0.9332(16)	0.064(4)
F7_9	0.171(2)	0.5248(15)	0.864(2)	0.109(7)
F8_9	0.237(2)	0.5696(18)	1.0281(17)	0.089(6)
F9_9	0.318(3)	0.6304(13)	0.8906(19)	0.098(6)
O1_10	0.476(3)	0.4183(17)	0.8633(19)	0.057(4)
C1_10	0.416(2)	0.4359(15)	0.9539(18)	0.058(4)
C2_10	0.528(3)	0.4752(19)	1.046(2)	0.072(5)
F1_10	0.653(3)	0.436(2)	1.035(3)	0.087(8)
F2_10	0.506(3)	0.446(2)	1.145(2)	0.082(7)
F3_10	0.527(4)	0.5637(18)	1.031(3)	0.086(7)
C3_10	0.287(2)	0.4954(17)	0.922(2)	0.070(5)
F4_10	0.178(3)	0.4450(19)	0.864(2)	0.102(9)
F5_10	0.318(3)	0.5628(17)	0.860(2)	0.072(6)

F6_10	0.240(3)	0.524(2)	1.012(2)	0.081(7)
C4_10	0.368(3)	0.3465(17)	0.992(2)	0.074(5)
F7_10	0.311(3)	0.2953(19)	0.9050(19)	0.075(7)
F8_10	0.269(4)	0.360(2)	1.055(3)	0.084(8)
F9_10	0.488(3)	0.3099(18)	1.047(2)	0.081(7)
O1_11	0.666(2)	0.3876(13)	0.727(2)	0.065(4)
C1_11	0.761(2)	0.3187(12)	0.7472(16)	0.058(4)
C2_11	0.879(3)	0.3325(18)	0.679(2)	0.091(6)
F1_11	0.994(2)	0.368(2)	0.747(3)	0.139(9)
F2_11	0.922(3)	0.2566(18)	0.641(3)	0.113(7)
F3_11	0.824(2)	0.3781(16)	0.590(2)	0.094(6)
C3_11	0.669(3)	0.2381(14)	0.7123(19)	0.070(4)
F4_11	0.549(3)	0.2400(17)	0.752(2)	0.099(6)
F5_11	0.634(3)	0.2455(17)	0.6018(16)	0.100(7)
F6_11	0.741(3)	0.1645(12)	0.743(2)	0.075(6)
C4_11	0.830(3)	0.3093(17)	0.8678(19)	0.085(5)
F7_11	0.734(3)	0.271(2)	0.921(2)	0.113(7)
F8_11	0.947(3)	0.259(2)	0.875(3)	0.096(8)
F9_11	0.863(4)	0.3890(15)	0.909(2)	0.114(9)
O1_12	0.630(2)	0.3663(12)	0.7974(18)	0.048(3)
C1_12	0.711(2)	0.2925(13)	0.7886(16)	0.057(4)
C2_12	0.817(3)	0.2740(16)	0.897(2)	0.077(5)
F1_12	0.879(4)	0.3498(17)	0.936(3)	0.109(9)
F2_12	0.925(3)	0.2223(18)	0.883(3)	0.079(8)
F3_12	0.741(3)	0.2423(16)	0.9685(19)	0.090(6)
C3_12	0.610(2)	0.2152(13)	0.7575(16)	0.049(4)
F4_12	0.539(2)	0.2223(19)	0.6517(15)	0.089(7)
F5_12	0.503(3)	0.2086(17)	0.815(2)	0.091(7)
F6_12	0.681(3)	0.1406(13)	0.769(2)	0.072(6)
C4_12	0.794(3)	0.3059(16)	0.695(2)	0.078(5)
F7_12	0.714(3)	0.337(2)	0.602(2)	0.110(7)
F8_12	0.855(3)	0.2301(14)	0.671(2)	0.090(6)
F9_12	0.898(3)	0.3638(19)	0.729(3)	0.121(7)

Table S 4: Bond lengths for and angles for $[Cu(C_2H_2)_3][Al(OR^F)_4]$ **1**.

Atom–Atom	Length [Å]
Cu1–C5	2.11(2)
Cu1–C1	2.12(2)
Cu1–C4	2.12(2)
Cu1–C2	2.13(2)
Cu1–C6	2.16(2)
Cu1–C3	2.18(2)
C1–C2	1.08(3)
C1–H1	0.95(3)
C2–H2	0.95(3)
C3–C4	1.06(3)
C3–H3	0.95(3)
C4–H4	0.96(3)
C5–C6	1.08(3)
C5–H5	0.95(3)
C6–H6	0.95(3)
Cu1A–C5A	2.12(3)
Cu1A–C2A	2.13(2)
Cu1A–C4A	2.13(2)
Cu1A–C1A	2.14(2)
Cu1A–C3A	2.17(3)
Cu1A–C6A	2.18(3)
C1A–C2A	1.09(3)
C1A–H1A	0.94(3)
C2A–H2A	0.95(3)
C3A–C4A	1.06(3)
C3A–H3A	0.95(3)
C4A–H4A	0.95(3)
C5A–C6A	1.07(3)
C5A–H5A	0.95(3)
C6A–H6A	0.95(3)
Al1–O1_11	1.650(19)
Al1–O1_7	1.665(17)
Al1–O1_12	1.669(16)
Al1–O1_6	1.67(2)
Al1–O1_9	1.680(17)
Al1–O1_8	1.711(18)
Al1–O1_10	1.75(2)
Al1–O1_5	1.76(2)
Al1–F9_6	2.35(3)

O1_5–C1_5	1.40(2)
C1_5–C2_5	1.50(2)
C1_5–C4_5	1.52(2)
C1_5–C3_5	1.53(2)
C2_5–F3_5	1.33(2)
C2_5–F2_5	1.34(2)
C2_5–F1_5	1.35(2)
C3_5–F6_5	1.33(2)
C3_5–F4_5	1.34(2)
C3_5–F5_5	1.34(2)
C4_5–F9_5	1.33(2)
C4_5–F8_5	1.33(2)
C4_5–F7_5	1.34(2)
O1_6–C1_6	1.42(2)
C1_6–C3_6	1.50(2)
C1_6–C4_6	1.51(2)
C1_6–C2_6	1.54(2)
C2_6–F2_6	1.32(2)
C2_6–F3_6	1.35(2)
C2_6–F1_6	1.35(2)
C3_6–F4_6	1.34(2)
C3_6–F5_6	1.34(2)
C3_6–F6_6	1.35(2)
C4_6–F7_6	1.32(2)
C4_6–F8_6	1.35(2)
C4_6–F9_6	1.38(2)
O1_7–C1_7	1.398(19)
C1_7–C2_7	1.50(2)
C1_7–C4_7	1.52(2)
C1_7–C3_7	1.53(2)
C2_7–F2_7	1.32(2)
C2_7–F3_7	1.33(2)
C2_7–F1_7	1.37(2)
C3_7–F4_7	1.33(2)
C3_7–F6_7	1.33(2)
C3_7–F5_7	1.34(2)
C4_7–F7_7	1.33(2)
C4_7–F9_7	1.34(2)
C4_7–F8_7	1.345(19)
O1_8–C1_8	1.38(2)
C1_8–C4_8	1.51(2)
C1_8–C2_8	1.52(2)
C1_8–C3_8	1.53(2)
C2_8–F3_8	1.32(2)
C2_8–F1_8	1.33(2)
C2_8–F2_8	1.35(2)
C3_8–F4_8	1.33(2)
C3_8–F5_8	1.33(2)
C3_8–F6_8	1.36(2)
C4_8–F9_8	1.33(2)
C4_8–F8_8	1.33(2)
C4_8–F7_8	1.36(2)
O1_9–C1_9	1.387(19)
C1_9–C3_9	1.50(2)
C1_9–C4_9	1.533(19)
C1_9–C2_9	1.533(19)
C2_9–F2_9	1.32(2)
C2_9–F1_9	1.34(2)
C2_9–F3_9	1.35(2)
C3_9–F4_9	1.313(19)
C3_9–F5_9	1.345(19)
C3_9–F6_9	1.362(19)
C4_9–F9_9	1.31(2)
C4_9–F7_9	1.330(19)
C4_9–F8_9	1.34(2)
O1_10–C1_10	1.37(2)
C1_10–C3_10	1.51(2)
C1_10–C2_10	1.53(2)
C1_10–C4_10	1.53(2)
C2_10–F2_10	1.34(2)
C2_10–F1_10	1.35(2)
C2_10–F3_10	1.36(2)
C3_10–F6_10	1.34(2)
C3_10–F5_10	1.34(2)
C3_10–F4_10	1.38(2)
C4_10–F9_10	1.33(2)
C4_10–F8_10	1.34(2)
C4_10–F7_10	1.35(2)
O1_11–C1_11	1.38(2)
C1_11–C4_11	1.51(2)
C1_11–C3_11	1.52(2)
C1_11–C2_11	1.53(2)
C2_11–F3_11	1.32(2)
C2_11–F2_11	1.33(2)
C2_11–F1_11	1.36(2)
C3_11–F4_11	1.32(2)
C3_11–F6_11	1.329(19)
C3_11–F5_11	1.34(2)
C4_11–F9_11	1.33(2)
C4_11–F8_11	1.33(2)
C4_11–F7_11	1.35(2)
O1_12–C1_12	1.38(2)
C1_12–C3_12	1.52(2)
C1_12–C4_12	1.52(2)
C1_12–C2_12	1.54(2)
C2_12–F3_12	1.33(2)
C2_12–F2_12	1.33(2)
C2_12–F1_12	1.34(2)
C3_12–F6_12	1.314(19)
C3_12–F5_12	1.35(2)
C3_12–F4_12	1.351(19)
C4_12–F7_12	1.33(2)
C4_12–F9_12	1.33(2)
C4_12–F8_12	1.35(2)

**Atom–Atom–Atom**	**Angle [°]**
C5–Cu1–C1	127.1(18)
C5–Cu1–C4	85.5(17)
C1–Cu1–C4	144.2(18)
C5–Cu1–C2	156.4(18)
C1–Cu1–C2	29.5(8)
C4–Cu1–C2	117.8(18)
C5–Cu1–C6	29.3(8)
C1–Cu1–C6	98.7(18)
C4–Cu1–C6	114.8(16)
C2–Cu1–C6	127.3(17)

C5–Cu1–C3	103.9(15)	O1_11–Al1–O1_9	140.8(11)
C1–Cu1–C3	128.7(18)	O1_7–Al1–O1_9	125.4(11)
C4–Cu1–C3	28.6(8)	O1_12–Al1–O1_8	129.5(12)
C2–Cu1–C3	99.2(18)	O1_6–Al1–O1_8	120.8(11)
C6–Cu1–C3	130.6(16)	O1_12–Al1–O1_10	69.8(11)
C2–C1–Cu1	76.1(16)	O1_6–Al1–O1_10	119.3(13)
C2–C1–H1	150(10)	O1_8–Al1–O1_10	104.1(13)
Cu1–C1–H1	134(10)	O1_11–Al1–O1_5	91.5(11)
C1–C2–Cu1	74.4(16)	O1_7–Al1–O1_5	96.0(13)
C1–C2–H2	147(10)	O1_9–Al1–O1_5	106.7(13)
Cu1–C2–H2	138(10)	O1_12–Al1–F9_6	175.0(10)
C4–C3–Cu1	72.7(16)	O1_6–Al1–F9_6	71.9(9)
C4–C3–H3	157(10)	O1_8–Al1–F9_6	54.2(10)
Cu1–C3–H3	130(10)	O1_10–Al1–F9_6	113.4(13)
C3–C4–Cu1	78.7(17)	C1_5–O1_5–Al1	154(2)
C3–C4–H4	149(10)	O1_5–C1_5–C2_5	107.3(19)
Cu1–C4–H4	130(10)	O1_5–C1_5–C4_5	108.2(18)
C6–C5–Cu1	77.6(16)	C2_5–C1_5–C4_5	115.3(18)
C6–C5–H5	151(10)	O1_5–C1_5–C3_5	102.6(19)
Cu1–C5–H5	131(10)	C2_5–C1_5–C3_5	110.4(17)
C5–C6–Cu1	73.1(16)	C4_5–C1_5–C3_5	112.0(17)
C5–C6–H6	146(10)	F3_5–C2_5–F2_5	101(2)
Cu1–C6–H6	137(10)	F3_5–C2_5–F1_5	111(2)
C5A–Cu1A–C2A	162.0(19)	F2_5–C2_5–F1_5	105(2)
C5A–Cu1A–C4A	101(2)	F3_5–C2_5–C1_5	113(2)
C2A–Cu1A–C4A	96.9(19)	F2_5–C2_5–C1_5	120(2)
C5A–Cu1A–C1A	132.4(18)	F1_5–C2_5–C1_5	107(2)
C2A–Cu1A–C1A	29.6(9)	F6_5–C3_5–F4_5	109(2)
C4A–Cu1A–C1A	126(2)	F6_5–C3_5–F5_5	112(2)
C5A–Cu1A–C3A	127.5(19)	F4_5–C3_5–F5_5	106(2)
C2A–Cu1A–C3A	70.3(19)	F6_5–C3_5–C1_5	106(2)
C4A–Cu1A–C3A	28.6(9)	F4_5–C3_5–C1_5	115(2)
C1A–Cu1A–C3A	99.4(19)	F5_5–C3_5–C1_5	110(2)
C5A–Cu1A–C6A	28.9(9)	F9_5–C4_5–F8_5	110(2)
C2A–Cu1A–C6A	133.1(18)	F9_5–C4_5–F7_5	108(2)
C4A–Cu1A–C6A	130.0(18)	F8_5–C4_5–F7_5	109(2)
C1A–Cu1A–C6A	103.5(19)	F9_5–C4_5–C1_5	108.9(19)
C3A–Cu1A–C6A	155.2(19)	F8_5–C4_5–C1_5	114(2)
C2A–C1A–Cu1A	74.8(16)	F7_5–C4_5–C1_5	108(2)
C2A–C1A–H1A	139(10)	C1_6–O1_6–Al1	139.3(16)
Cu1A–C1A–H1A	146(10)	O1_6–C1_6–C3_6	105.5(17)
C1A–C2A–Cu1A	75.6(16)	O1_6–C1_6–C4_6	104.8(18)
C1A–C2A–H2A	136(10)	C3_6–C1_6–C4_6	118.8(19)
Cu1A–C2A–H2A	148(10)	O1_6–C1_6–C2_6	97.5(18)
C4A–C3A–Cu1A	73.7(17)	C3_6–C1_6–C2_6	113.8(18)
C4A–C3A–H3A	142(10)	C4_6–C1_6–C2_6	113.2(18)
Cu1A–C3A–H3A	144(10)	F2_6–C2_6–F3_6	111(2)
C3A–C4A–Cu1A	77.7(17)	F2_6–C2_6–F1_6	110(2)
C3A–C4A–H4A	140(10)	F3_6–C2_6–F1_6	98(2)
Cu1A–C4A–H4A	142(10)	F2_6–C2_6–C1_6	113(2)
C6A–C5A–Cu1A	78.4(17)	F3_6–C2_6–C1_6	116(2)
C6A–C5A–H5A	139(10)	F1_6–C2_6–C1_6	109(2)
Cu1A–C5A–H5A	142(10)	F4_6–C3_6–F5_6	100(2)
C5A–C6A–Cu1A	72.7(16)	F4_6–C3_6–F6_6	109(3)
C5A–C6A–H6A	136(10)	F5_6–C3_6–F6_6	104(2)
Cu1A–C6A–H6A	151(10)	F4_6–C3_6–C1_6	117(2)
O1_11–Al1–O1_7	85.2(12)	F5_6–C3_6–C1_6	113(2)
O1_12–Al1–O1_6	103.4(12)	F6_6–C3_6–C1_6	111(2)

F7_6–C4_6–F8_6	112(3)
F7_6–C4_6–F9_6	92(2)
F8_6–C4_6–F9_6	99(3)
F7_6–C4_6–C1_6	120(2)
F8_6–C4_6–C1_6	117(2)
F9_6–C4_6–C1_6	112(2)
C4_6–F9_6–Al1	110.1(18)
C1_7–O1_7–Al1	152.3(19)
O1_7–C1_7–C2_7	115.7(18)
O1_7–C1_7–C4_7	103.9(16)
C2_7–C1_7–C4_7	109.9(16)
O1_7–C1_7–C3_7	106.9(17)
C2_7–C1_7–C3_7	108.6(16)
C4_7–C1_7–C3_7	111.9(17)
F2_7–C2_7–F3_7	112(2)
F2_7–C2_7–F1_7	103(2)
F3_7–C2_7–F1_7	109(2)
F2_7–C2_7–C1_7	114(2)
F3_7–C2_7–C1_7	108.8(19)
F1_7–C2_7–C1_7	110(2)
F4_7–C3_7–F6_7	111(3)
F4_7–C3_7–F5_7	108(2)
F6_7–C3_7–F5_7	110(3)
F4_7–C3_7–C1_7	103.2(19)
F6_7–C3_7–C1_7	115(2)
F5_7–C3_7–C1_7	109(2)
F7_7–C4_7–F9_7	106(2)
F7_7–C4_7–F8_7	116(2)
F9_7–C4_7–F8_7	103(2)
F7_7–C4_7–C1_7	105.8(18)
F9_7–C4_7–C1_7	114(2)
F8_7–C4_7–C1_7	112(2)
C1_8–O1_8–Al1	151.1(18)
O1_8–C1_8–C4_8	105.0(17)
O1_8–C1_8–C2_8	110.7(17)
C4_8–C1_8–C2_8	110.4(16)
O1_8–C1_8–C3_8	114.3(18)
C4_8–C1_8–C3_8	107.3(16)
C2_8–C1_8–C3_8	109.0(16)
F3_8–C2_8–F1_8	108(2)
F3_8–C2_8–F2_8	105(2)
F1_8–C2_8–F2_8	102(2)
F3_8–C2_8–C1_8	112.1(19)
F1_8–C2_8–C1_8	118(2)
F2_8–C2_8–C1_8	111.3(19)
F4_8–C3_8–F5_8	106(2)
F4_8–C3_8–F6_8	107(3)
F5_8–C3_8–F6_8	107(3)
F4_8–C3_8–C1_8	111(2)
F5_8–C3_8–C1_8	117(2)
F6_8–C3_8–C1_8	109(2)
F9_8–C4_8–F8_8	106(2)
F9_8–C4_8–F7_8	101(2)
F8_8–C4_8–F7_8	111(2)
F9_8–C4_8–C1_8	118(2)
F8_8–C4_8–C1_8	111(2)
F7_8–C4_8–C1_8	109.5(19)
C1_9–O1_9–Al1	152.8(17)
O1_9–C1_9–C3_9	110.3(15)
O1_9–C1_9–C4_9	111.3(15)
C3_9–C1_9–C4_9	110.7(14)
O1_9–C1_9–C2_9	103.5(16)
C3_9–C1_9–C2_9	111.6(15)
C4_9–C1_9–C2_9	109.3(14)
F2_9–C2_9–F1_9	112(2)
F2_9–C2_9–F3_9	102.9(18)
F1_9–C2_9–F3_9	108.0(19)
F2_9–C2_9–C1_9	111.2(17)
F1_9–C2_9–C1_9	108.8(16)
F3_9–C2_9–C1_9	114.0(17)
F4_9–C3_9–F5_9	107.3(18)
F4_9–C3_9–F6_9	102(2)
F5_9–C3_9–F6_9	107.1(18)
F4_9–C3_9–C1_9	118.3(17)
F5_9–C3_9–C1_9	109.4(16)
F6_9–C3_9–C1_9	112.0(17)
F9_9–C4_9–F7_9	107(2)
F9_9–C4_9–F8_9	111(2)
F7_9–C4_9–F8_9	104(2)
F9_9–C4_9–C1_9	109.9(17)
F7_9–C4_9–C1_9	113.9(17)
F8_9–C4_9–C1_9	110.3(18)
C1_10–O1_10–Al1	145(2)
O1_10–C1_10–C3_10	110.4(18)
O1_10–C1_10–C2_10	109.7(19)
C3_10–C1_10–C2_10	112.1(17)
O1_10–C1_10–C4_10	105.2(18)
C3_10–C1_10–C4_10	110.1(17)
C2_10–C1_10–C4_10	109.1(17)
F2_10–C2_10–F1_10	103(2)
F2_10–C2_10–F3_10	117(2)
F1_10–C2_10–F3_10	114(2)
F2_10–C2_10–C1_10	110(2)
F1_10–C2_10–C1_10	105(2)
F3_10–C2_10–C1_10	107(2)
F6_10–C3_10–F5_10	111(2)
F6_10–C3_10–F4_10	106(2)
F5_10–C3_10–F4_10	111(2)
F6_10–C3_10–C1_10	111(2)
F5_10–C3_10–C1_10	110.7(19)
F4_10–C3_10–C1_10	107.0(19)
F9_10–C4_10–F8_10	113(2)
F9_10–C4_10–F7_10	110(2)
F8_10–C4_10–F7_10	109(2)
F9_10–C4_10–C1_10	104.7(19)
F8_10–C4_10–C1_10	108(2)
F7_10–C4_10–C1_10	112(2)
C1_11–O1_11–Al1	155.9(19)
O1_11–C1_11–C4_11	113.4(17)
O1_11–C1_11–C3_11	104.0(16)
C4_11–C1_11–C3_11	108.7(16)
O1_11–C1_11–C2_11	108.6(17)
C4_11–C1_11–C2_11	108.8(16)
C3_11–C1_11–C2_11	113.4(17)
F3_11–C2_11–F2_11	106(2)
F3_11–C2_11–F1_11	117(2)

F2_11–C2_11–F1_11	107(2)
F3_11–C2_11–C1_11	107.9(19)
F2_11–C2_11–C1_11	112(2)
F1_11–C2_11–C1_11	108(2)
F4_11–C3_11–F6_11	110(2)
F4_11–C3_11–F5_11	108(2)
F6_11–C3_11–F5_11	112(2)
F4_11–C3_11–C1_11	112(2)
F6_11–C3_11–C1_11	111.3(19)
F5_11–C3_11–C1_11	103.8(18)
F9_11–C4_11–F8_11	111(2)
F9_11–C4_11–F7_11	110(2)
F8_11–C4_11–F7_11	111(2)
F9_11–C4_11–C1_11	108.2(19)
F8_11–C4_11–C1_11	109(2)
F7_11–C4_11–C1_11	107.9(19)
C1_12–O1_12–Al1	155.9(15)
O1_12–C1_12–C3_12	108.7(16)
O1_12–C1_12–C4_12	108.9(16)
C3_12–C1_12–C4_12	107.7(16)
O1_12–C1_12–C2_12	111.5(16)
C3_12–C1_12–C2_12	110.8(15)
C4_12–C1_12–C2_12	109.1(17)
F3_12–C2_12–F2_12	113(2)
F3_12–C2_12–F1_12	110(2)
F2_12–C2_12–F1_12	105(2)
F3_12–C2_12–C1_12	106.8(19)
F2_12–C2_12–C1_12	113.3(19)
F1_12–C2_12–C1_12	109(2)
F6_12–C3_12–F5_12	107(2)
F6_12–C3_12–F4_12	109(2)
F5_12–C3_12–F4_12	102(2)
F6_12–C3_12–C1_12	111.0(18)
F5_12–C3_12–C1_12	115.3(18)
F4_12–C3_12–C1_12	110.9(18)
F7_12–C4_12–F9_12	108(3)
F7_12–C4_12–F8_12	109(2)
F9_12–C4_12–F8_12	108(2)
F7_12–C4_12–C1_12	113.3(19)
F9_12–C4_12–C1_12	108(2)
F8_12–C4_12–C1_12	110.6(19)

5. Computational details

General Information

Gibbs free energies $G°$ for molecular structures were computed at T = 298.15 K and p = 1.00 atm. The Gibbs free energy can be calculated using Eq. S4.

$$G° = H° - T \cdot S° \tag{S4}$$

While $G°$ can directly be extracted from the ORCA output-file for polyatomic structures, it has to be calculated for monoatomic structures using Eq. S4 and Eq. S5, as well as the Sackur-Tetrode equation for the determination of the standard entropy S°.[36]

$$H° = E_{el} + E_{vrt} + R \cdot T \tag{S5}$$

In Eq. S5, E_{el} is the electronic energy (found as 'final single point energy' in the ORCA output-file), E_{vrt} is the sum of translational, rotational and vibrational energies including zero-point energy (for monoatomic structures $E_{vrt} = \frac{3}{2}RT$ resulting from the translational contribution) and R is the ideal gas constant.

Table S 5: Summary of computed thermodynamic data for monoatomic structures at the RI-r^{2}SCAN-3c(D4)/def2-mTZVPP, RIJCOSX-B3LYP(D4)/def2-TZVPP and DLPNO-CCSD(T)/def2-QZVPP//RI-r^{2}SCAN-3c(D4)/def2-mTZVPP levels of theory.

Atom	Method	E_{el} / kJ mol^{-1}	$H°$ / kJ mol^{-1}	$G°$ / kJ mol^{-1}	$S°$ / kJ mol^{-1} K^{-1}
Cu^+	r^{2}SCAN-3c	−4306265.17	−4306258.97	−4306306.84	0.1606
	B3LYP	−4306174.39	−4306168.20	−4306216.07	0.1606
	DLPNO-CCSD(T)	−4304386.60	−4304380.40	−4304428.27	0.1606
Ag^+	r^{2}SCAN-3c	−385230.74	−385224.54	−385274.38	0.1672
	B3LYP	−385081.29	−385075.10	−385124.94	0.1672
	DLPNO-CCSD(T)	−384795.88	−384789.69	−384839.52	0.1672

Table S 6: Summary of computed thermodynamic data for polyatomic structures at the RI-r^{2}SCAN-3c(D4)/def2-mTZVPP, RIJCOSX-B3LYP(D4)/def2-TZVPP and DLPNO-CCSD(T)/def2-QZVPP//RI-r^{2}SCAN-3c(D4)/def2-mTZVPP levels of theory.

Molecule	$G°$ / kJ mol^{-1} r^{2}SCAN-3c	$G°$ / kJ mol^{-1} B3LYP	$G°$ / kJ mol^{-1} DLPNO-CCSD(T)
C_2H_2	−202936.10	−202971.24	−202689.67
$[Cu(C_2H_2)]^+$	−4509441.85	−4509353.92	−4507254.41
$[Cu(C_2H_2)_2]^+$	−4712543.93	−4712460.56	−4710074.10
$[Cu(C_2H_2)_3]^+$	−4915529.22	−4915461.66	−4912789.40
$[Cu(C_2H_2)_3]^+$ (C_2)		−4915425.981	−4912759.306
$[Cu(C_2H_2)_4]^+$	−5118461.09	−5118426.09	−5115471.90
$[Ag(C_2H_2)]^+$	−588346.97	−588217.90	−587633.43
$[Ag(C_2H_2)_2]^+$	−791398.57	−791285.80	−790411.72
$[Ag(C_2H_2)_3]^+$	−994362.00	−994275.51	−993116.26
$[Ag(C_2H_2)_4]^+$	−1197304.77	−1197248.99	−1195813.27

Table S 7: Computed ^1H and ^{13}C isotropic chemical shifts for free C_2H_2 and **1**$^+$ (RIJCOSX-B3LYP(D4)/def2-TZVPP).

Molecule	$\delta_{H(1)}$ / ppm	$\delta_{H(2)}$ / ppm	$\delta_{C(1)}$ / ppm	$\delta_{C(2)}$ / ppm
C_2H_2	30.5	30.5	112	112
$[Cu(C_2H_2)_3]^+$	27.9	27.9	103	103

EDA-NOCV Analysis: General Information

In the EDA-NOCV[31] (energy decomposition analysis – natural orbitals for chemical valence) analysis, the interaction energy during bond formation ΔE_{int} is decomposed into four components:

$$\Delta E_{int} = \Delta E_{elstat} + \Delta E_{Pauli} + \Delta E_{orb} + \Delta E_{disp} \qquad (S6)$$

with ΔE_{elstat} being the electrostatic interaction, ΔE_{Pauli} the 'exchange repulsion' arising from the Pauli exclusion principle and change in the exchange-correlation energy (ΔE_{Pauli} ist further subdivided into $\Delta E_{Pauli} = \Delta\tilde{E}_{Pauli} + \Delta E_{XC}^{0}$), ΔE_{orb} the orbital interaction and ΔE_{disp} the dispersion interaction.

The orbital interaction is further decomposed into contributions associated with orbital pairs of fragments, which have to be defined first. These contributions can be written as addends making up the entire orbital interaction. In this work, only the first and second addends of this sum are regarded, as they make up together ca. 80 % of the entire orbital contribution.

EDA-NOCV Analysis of $[Cu(C_2H_2)_3]^+$

Defined Fragments: $[Cu(C_2H_2)_2]^+$ (charge 1, multiplicity 1) + C_2H_2 (charge 0, multiplicity 1)

EDA-NOCV Analysis of $[Cu(C_2H_2)_2]^+$

Defined Fragments: $[Cu(C_2H_2)]^+$ (charge 1, multiplicity 1) + C_2H_2 (charge 0, multiplicity 1)

Table S 8: Summary of results obtained by EDA-NOCV analysis (RIJCOSX-B3LYP(D4)/def2-TZVPP). The first contribution to the orbital interaction corresponds in both molecules to σ-donation, the second one to π-backdonation.

Energy Term	$[Cu(C_2H_2)_3]^+$ Energy / kcal/mol	$[Cu(C_2H_2)_2]^+$ Energy / kcal/mol
ΔE_{int}	−28.71	−43.40
ΔE_{elstat}	−56.01	−65.06
$\Delta\tilde{E}_{Pauli}$	109.04	107.78
ΔE_{XC}^{0}	−43.37	−41.93
ΔE_{disp}	−4.00	−2.32
ΔE_{orb} (total)	−34.35	−41.75
ΔE_{orb} (1st contribution)	−17.68 (51 %)	−20.17 (48 %)
ΔE_{orb} (2nd contribution)	−9.62 (28 %)	−11.31 (27 %)
ΔE_{orb} (3rd contribution)	−2.74 (8 %)	−4.71 (11 %)
ΔE_{orb} (4th contribution)	−2.28 (7 %)	−2.64 (6 %)

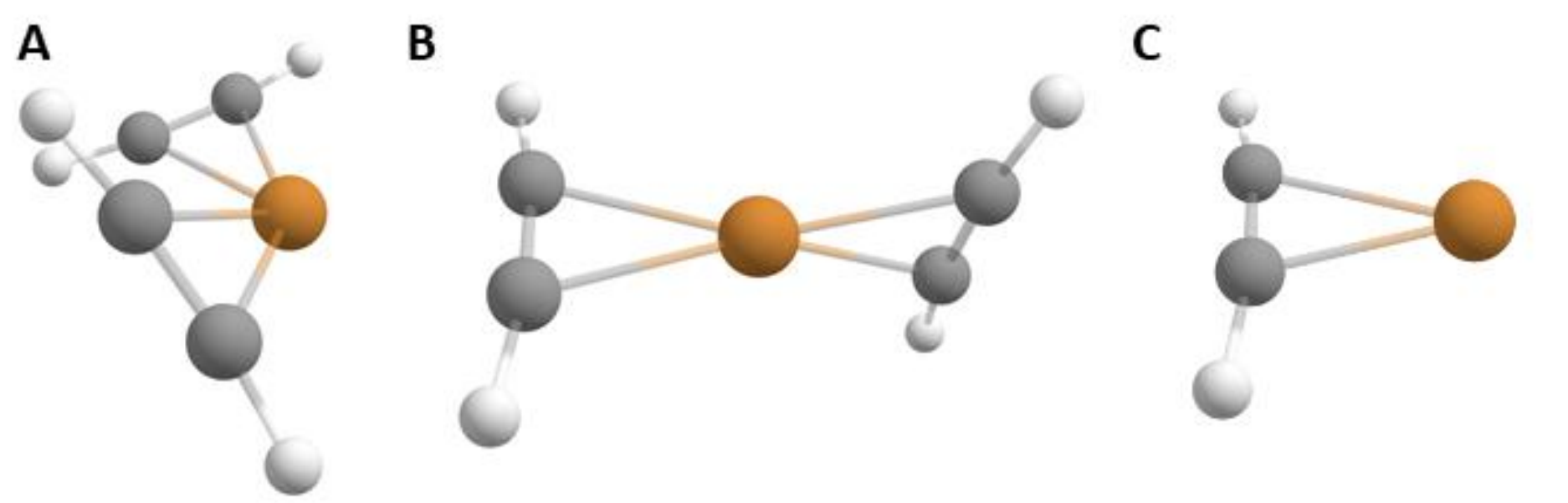

Figure S 10: A) Fragment used for the EDA-NOCV analysis of $[Cu(C_2H_2)_2]^+$ + C_2H_2, B) optimized minimum geometry for $[Cu(C_2H_2)_2]^+$ and C) structure of $[Cu(C_2H_2)]^+$. Structure A is 43 kJ mol^{-1} higher in energy than structure B (RIJCOSX-B3LYP(D4)/def2-TZVPP).

Coordinates of computed structures at the RI-r^{2}SCAN-3c(D4)/def2-mTZVPP level of theory in Angstrom

C_2H_2

C	-2.381746	0.671359	0.093301
C	-1.181247	0.680762	0.094606
H	-3.445355	0.652042	0.090615
H	-0.117691	0.698787	0.097109

$[Cu(C_2H_2)]^+$

Cu	3.723721	10.807297	24.389178
C	3.628703	8.796090	24.519732
H	4.177067	8.173998	23.833372
C	2.908690	9.262896	25.400513
H	2.247328	9.437639	26.231685

$[Cu(C_2H_2)_2]^+$

Cu	2.919834	10.731629	24.828761
C	3.643466	8.849215	24.565892
H	4.024047	8.562648	23.602554
C	3.262992	8.939779	25.727240
H	3.002300	8.812845	26.762090
C	2.949972	12.759605	24.675471
H	3.848589	13.292346	24.927771
C	1.832407	12.380670	24.345162
H	0.814701	12.262413	24.020669

$[Cu(C_2H_2)_3]^+$

Cu	3.744812	10.889790	24.362859
C	3.621113	8.776677	24.529199
H	4.166659	8.157291	23.846833
C	2.904741	9.240032	25.403825
H	2.248195	9.414132	26.231766
C	5.243668	11.728702	23.112475
H	5.023199	12.605778	22.538857
C	5.735118	10.739352	23.634246
H	6.364609	9.941747	23.972521
C	2.946996	12.827609	24.727071
H	3.605169	13.672080	24.748949
C	2.006766	12.048714	24.768684
H	1.053865	11.564826	24.836426

$[Cu(C_2H_2)_4]^+$

Cu	14.022009	-7.010999	7.200310
C	15.091218	-5.298537	8.237331
H	16.053914	-5.056060	7.841221
C	14.034775	-5.441981	8.818843
H	13.160725	-5.460347	9.434847
C	12.952766	-8.723466	8.237308
H	11.990033	-8.965854	7.841234
C	14.009137	-8.579930	8.818927
H	14.883201	-8.561621	9.434912
C	15.734449	-8.080264	6.163311
H	15.976877	-9.042970	6.559428
C	15.591029	-7.023836	5.581768
H	15.572687	-6.149797	4.965747
C	12.309574	-5.941782	6.163220
H	12.067138	-4.979060	6.559291
C	12.453047	-6.998215	5.581698
H	12.471430	-7.872271	4.965704

$[Ag(C_2H_2)]^+$

Ag	3.775296	11.008887	24.324305
C	3.604902	8.718622	24.547803
H	4.174549	8.150621	23.836969
C	2.892390	9.181988	25.423721
H	2.238373	9.417812	26.241672

$[Ag(C_2H_2)_2]^+$

Ag	2.920388	10.732034	24.829080
C	3.712550	8.608339	24.610586
H	4.086625	8.357730	23.637225
C	3.333061	8.697829	25.766170
H	3.058296	8.605575	26.798722
C	2.877756	13.000407	24.632133
H	3.791684	13.499005	24.889616
C	1.765274	12.622919	24.304628
H	0.752676	12.467311	23.987440

$[Ag(C_2H_2)_3]^+$

Ag	3.743891	10.889314	24.362802
C	3.553893	8.531649	24.607648
H	4.115391	7.967000	23.893236
C	2.848830	8.993083	25.484641
H	2.204241	9.229666	26.305012
C	5.472412	11.769898	22.984300
H	5.194638	12.646223	22.437156
C	5.962301	10.786888	23.506426
H	6.541862	9.969811	23.881859
C	2.779527	13.026691	24.777414
H	3.484292	13.831444	24.788273
C	1.843322	12.251521	24.814787

H	0.920309	11.713542	24.870156

$[Ag(C_2H_2)_4]^+$

Ag	14.021982	-7.010985	7.200298
C	15.156705	-5.102255	8.392885
H	16.089667	-4.869172	7.927485
C	14.129539	-5.270062	9.011347
H	13.262283	-5.339710	9.631716
C	12.887305	-8.919801	8.392832
H	11.954359	-9.152914	7.927415
C	13.914360	-8.751813	9.011426
H	14.781612	-8.682148	9.631799
C	15.930778	-8.145713	6.007785
H	16.163900	-9.078633	6.473248
C	15.762918	-7.118598	5.389253
H	15.693247	-6.251371	4.768847
C	12.113260	-5.876299	6.007667
H	11.880128	-4.943352	6.473072
C	12.281143	-6.903441	5.389186
H	12.350804	-7.770712	4.768840

Coordinates of computed structures at the RIJCOSX-B3LYP(D4)/def2-TZVPP level of theory in Angstrom

C_2H_2

C	-2.379902	0.670767	0.093218
C	-1.183126	0.681148	0.094659
H	-3.441992	0.652361	0.090660
H	-0.121020	0.698675	0.097093

$[Cu(C_2H_2)]^+$

Cu	3.733041	10.844755	24.377418
C	3.615262	8.761038	24.534853
H	4.183352	8.191447	23.825872
C	2.903609	9.223244	25.409479
H	2.250245	9.457436	26.226859

$[Cu(C_2H_2)_2]^+$

Cu	2.919815	10.731463	24.825870
C	3.655899	8.786869	24.579383
H	4.020816	8.535430	23.604716
C	3.286237	8.882406	25.733765
H	3.018638	8.798031	26.767064
C	2.926282	12.818739	24.668784
H	3.838236	13.311965	24.936122
C	1.820112	12.442695	24.332179
H	0.812275	12.283550	24.007727

$[Cu(C_2H_2)_3]^+$ (D_3)

Cu	3.743161	10.892234	24.363843
C	3.571073	8.716254	24.523440
H	4.082572	8.152207	23.775243
C	2.923277	9.170867	25.441747
H	2.326392	9.395757	26.297715
C	5.301694	11.716427	23.063489
H	5.020082	12.560494	22.473799
C	5.804576	10.771341	23.632102
H	6.399342	9.986754	24.044660
C	2.898507	12.876397	24.760057
H	3.599317	13.680135	24.807317
C	1.961499	12.107414	24.754960
H	1.033418	11.580448	24.775339

$[Cu(C_2H_2)_3]^+$ (C_2)

Cu	3.744650	10.890410	24.362730
C	3.341680	8.667010	24.207090
H	2.938770	8.245520	23.309140
C	3.806120	8.887680	25.315330
H	4.222820	8.846420	26.300730
C	5.456920	11.923730	23.730460
H	5.436300	12.993990	23.753470
C	5.734170	10.738380	23.628050
H	6.198070	9.781570	23.503120
C	2.947560	12.826640	24.734590
H	3.605540	13.608020	24.413720
C	2.035090	12.138740	25.166590
H	1.121950	11.741280	25.559810

$[Cu(C_2H_2)_4]^+$

Cu	14.022009	-7.010997	7.200309
C	15.109316	-5.232455	8.286166
H	16.061183	-5.014973	7.858737
C	14.062643	-5.376705	8.869463
H	13.180440	-5.419544	9.466677
C	12.934670	-8.789550	8.286144
H	11.982793	-9.006990	7.858715
C	13.981273	-8.645205	8.869543
H	14.863472	-8.602392	9.466765
C	15.800536	-8.098355	6.114479
H	16.017990	-9.050219	6.541931
C	15.656298	-7.051702	5.531143
H	15.613472	-6.169516	4.933904

C	12.243491	-5.923683	6.114383
H	12.026030	-4.971800	6.541787
C	12.387770	-6.970348	5.531079
H	12.430624	-7.852556	4.933875

$[Ag(C_2H_2)]^+$

Ag	3.784166	11.043670	24.313127
C	3.597767	8.700293	24.556201
H	4.173654	8.153932	23.838105
C	2.889692	9.160657	25.428219
H	2.240232	9.419378	26.238817

$[Ag(C_2H_2)_2]^+$

Ag	2.922970	10.732497	24.827266
C	3.722777	8.551263	24.621294
H	4.083999	8.319154	23.642034
C	3.353204	8.646769	25.772425
H	3.077740	8.581750	26.803587
C	2.857289	13.054719	24.627227
H	3.775680	13.531688	24.896284
C	1.754326	12.677464	24.292695
H	0.750326	12.495845	23.972788

$[Ag(C_2H_2)_3]^+$

Ag	3.742670	10.891404	24.363553
C	3.496681	8.466171	24.601269
H	4.002652	7.938194	23.824450
C	2.872525	8.920478	25.531344
H	2.300718	9.188047	26.391358
C	5.529114	11.754415	22.924217
H	5.202032	12.585809	22.341205
C	6.038645	10.824874	23.504871
H	6.606244	10.039889	23.951902
C	2.731057	13.076837	24.824739
H	3.465915	13.847446	24.890240
C	1.792954	12.315504	24.788934
H	0.883702	11.757662	24.775629

$[Ag(C_2H_2)_4]^+$

Ag	14.021984	-7.010984	7.200300
C	15.173165	-5.043707	8.424537
H	16.090765	-4.816691	7.932650
C	14.162292	-5.219738	9.055640
H	13.301075	-5.308891	9.676934
C	12.870840	-8.978332	8.424490
H	11.953258	-9.205378	7.932585
C	13.881618	-8.802145	9.055704
H	14.742824	-8.712977	9.677011
C	15.989319	-8.162174	5.976138
H	16.216368	-9.079736	6.468082
C	15.813241	-7.151349	5.344970
H	15.724069	-6.290164	4.723635
C	12.054714	-5.859844	5.976013
H	11.827650	-4.942255	6.467899
C	12.230819	-6.870696	5.344896
H	12.319992	-7.731921	4.723617

Computed Raman spectrum of $[Cu(C_2H_2)_3]^+$ at the RI-r²SCAN-3c(D4)/def2-mTZVPP level of theory

Mode	freq (cm**-1)	Activity	Depolarization
6:	42.63	1.368134	0.749999
7:	52.46	1.200003	0.749891
8:	118.79	6.945894	0.749526
9:	134.73	0.000935	0.749076
10:	157.93	1.187884	0.749964
11:	158.91	1.122188	0.749994
12:	284.46	0.659534	0.748688
13:	285.22	0.691340	0.749351
14:	291.54	2.122902	0.035504
15:	323.09	3.279170	0.749994
16:	326.21	14.681398	0.750000
17:	328.64	15.461890	0.749998
18:	623.39	1.135795	0.749882
19:	623.70	1.051602	0.749863
20:	630.36	1.461647	0.718235
21:	678.92	0.001416	0.718147
22:	685.32	0.031132	0.736708
23:	686.84	0.029111	0.748311
24:	763.02	0.034815	0.748279
25:	766.28	1.222064	0.733042
26:	768.01	1.291246	0.749654
27:	795.46	7.758527	0.749044
28:	797.34	7.791425	0.741204
29:	813.72	24.374666	0.117716
30:	1947.57	222.496910	0.046023
31:	1948.11	63.550856	0.107712
32:	1948.93	41.738178	0.160024
33:	3352.07	0.334296	0.747410
34:	3354.15	42.759587	0.749990
35:	3354.63	42.995699	0.749997
36:	3440.99	20.688455	0.737853
37:	3441.14	19.990123	0.749135
38:	3444.08	101.254108	0.022759

6. References

[1] J. Willrett, M. Schmitt, V. Zhuravlev, M. Sellin, P. J. Malinowski and I. Krossing, "Synthesis and Characterization of a Copper Dinitrogen Complex Supported by a Weakly Coordinating Anion," Angew. Chem. Int. Ed. 63, no. 38 (2024): e202405330. https://doi.org/10.1002/anie.202405330.

[2] R. K. Harris, E. D. Becker, S. M. Cabral de Menezes, R. Goodfellow and P. Granger, "NMR nomenclature: nuclear spin properties and conventions for chemical shifts. IUPAC Recommendations 2001. International Union of Pure and Applied Chemistry. Physical Chemistry Division. Commission on Molecular Structure and Spectroscopy," *Magn. Reson. Chem.* 40, no. 7 (2002): 489. https://doi.org/10.1002/mrc.1042.

[3] J. Cosier and A. M. Glazer, "A nitrogen-gas-stream cryostat for general X-ray diffraction studies," *J. Appl. Crystallogr.* 19, no. 2 (1986): 105. https://doi.org/10.1107/S0021889886089835.

[4] Bruker, *SAINT*, *V 8.40B*. Bruker AXS Inc., Madison, Wisconsin, USA.

[5] Bruker, *SADABS*. Bruker AXS Inc., Madison, Wisconsin, USA.

[6] G. M. Sheldrick, "SHELXT - integrated space-group and crystal-structure determination," *Acta Crystallogr., Sect. A: Found. Crystallogr.* 71, no. Pt 1 (2015): 3. https://doi.org/10.1107/S2053273314026370.

[7] G. M. Sheldrick, "Crystal structure refinement with SHELXL," *Acta Crystallogr., Sect. C: Cryst. Struct. Commun.* 71, no. Pt 1 (2015): 3. https://doi.org/10.1107/S2053229614024218.

[8] C. B. Hübschle, G. M. Sheldrick and B. Dittrich, "ShelXle: a Qt graphical user interface for SHELXL," *J. Appl. Crystallogr.* 44, no. Pt 6 (2011): 1281. https://doi.org/10.1107/S0021889811043202.

[9] D. Kratzert, J. J. Holstein and I. Krossing, "DSR: enhanced modelling and refinement of disordered structures with SHELXL," *J. Appl. Crystallogr.* 48, no. Pt 3 (2015): 933. https://doi.org/10.1107/S1600576715005580.

[10] D. Kratzert and I. Krossing, "Recent improvements in DSR," *J. Appl. Crystallogr.* 51, no. 3 (2018): 928. https://doi.org/10.1107/S1600576718004508.

[11] D. Kratzert, FinalCif, V73. https://www.xs3.uni-freiburg.de/research/finalcif.

[12] C. R. Groom, I. J. Bruno, M. P. Lightfoot and S. C. Ward, "The Cambridge Structural Database," *Acta Crystallogr., Sect. B: Struct. Sci* 72, no. Pt 2 (2016): 171. https://doi.org/10.1107/S2052520616003954.

[13] F. Neese, F. Wennmohs, U. Becker and C. Riplinger, "The ORCA quantum chemistry program package," *J. Chem. Phys.* 152, no. 22 (2020): 224108. https://doi.org/10.1063/5.0004608.

[14] F. Neese, "Software Update: The ORCA Program System—Version 6.0," *WIREs Comput. Mol. Sci.* 15, no. 2 (2025): e70019. https://doi.org/10.1002/wcms.70019.

[15] S. Grimme, A. Hansen, S. Ehlert and J.-M. Mewes, "r2SCAN-3c: A "Swiss army knife" composite electronic-structure method," *J. Chem. Phys.* 154, no. 6 (2021): 64103. https://doi.org/10.1063/5.0040021.

[16] F. Weigend and R. Ahlrichs, "Balanced basis sets of split valence, triple zeta valence and quadruple zeta valence quality for H to Rn: Design and assessment of accuracy," *Phys. Chem. Chem. Phys.* 7, no. 18 (2005): 3297. https://doi.org/10.1039/b508541a.

[17] F. Weigend, "Accurate Coulomb-fitting basis sets for H to Rn," *Phys. Chem. Chem. Phys.* 8, no. 9 (2006): 1057. https://doi.org/10.1039/b515623h.

[18] M. Sierka, A. Hogekamp and R. Ahlrichs, "Fast evaluation of the Coulomb potential for electron densities using multipole accelerated resolution of identity approximation," *J. Chem. Phys.* 118, no. 20 (2003): 9136. https://doi.org/10.1063/1.1567253.

[19] R. Ahlrichs, "Efficient evaluation of three-center two-electron integrals over Gaussian functions," *Phys. Chem. Chem. Phys.* 6, no. 22 (2004): 5119. https://doi.org/10.1039/b413539c.

[20] E. Caldeweyher, C. Bannwarth and S. Grimme, "Extension of the D3 dispersion coefficient model," *J. Chem. Phys.* 147, no. 3 (2017): 34112. https://doi.org/10.1063/1.4993215.
[21] A. D. Becke, "A new mixing of Hartree–Fock and local density-functional theories," *J. Chem. Phys.* 98, no. 2 (1993): 1372. https://doi.org/10.1063/1.464304.
[22] A. D. Becke, "Density-functional thermochemistry. III. The role of exact exchange," *J. Chem. Phys.* 98, no. 7 (1993): 5648. https://doi.org/10.1063/1.464913.
[23] C. Lee, W. Yang and R. G. Parr, "Development of the Colle-Salvetti correlation-energy formula into a functional of the electron density," *Phys. Rev. B: Condens. Matter* 37, no. 2 (1988): 785. https://doi.org/10.1103/physrevb.37.785.
[24] F. Neese, "An improvement of the resolution of the identity approximation for the formation of the Coulomb matrix," *J. comput. chem.* 24, no. 14 (2003): 1740. https://doi.org/10.1002/jcc.10318.
[25] F. Neese, F. Wennmohs, A. Hansen and U. Becker, "Efficient, approximate and parallel Hartree–Fock and hybrid DFT calculations. A 'chain-of-spheres' algorithm for the Hartree–Fock exchange," *Chem. Phys.* 356, no. 1-3 (2009): 98. https://doi.org/10.1016/j.chemphys.2008.10.036.
[26] B. Helmich-Paris, B. de Souza, F. Neese and R. Izsák, "An improved chain of spheres for exchange algorithm," *J. Chem. Phys.* 155, no. 10 (2021): 104109. https://doi.org/10.1063/5.0058766.
[27] E. Caldeweyher, S. Ehlert and A. Hansen et al., "A generally applicable atomic-charge dependent London dispersion correction," *J. Chem. Phys.* 150, no. 15 (2019): 154122. https://doi.org/10.1063/1.5090222.
[28] C. Riplinger, B. Sandhoefer, A. Hansen and F. Neese, "Natural triple excitations in local coupled cluster calculations with pair natural orbitals," *J. Chem. Phys.* 139, no. 13 (2013): 134101. https://doi.org/10.1063/1.4821834.
[29] D. G. Liakos, M. Sparta, M. K. Kesharwani, J. M. L. Martin and F. Neese, "Exploring the Accuracy Limits of Local Pair Natural Orbital Coupled-Cluster Theory," *J. Chem. Theory Comput.* 11, no. 4 (2015): 1525. https://doi.org/10.1021/ct501129s.
[30] F. Weigend, F. Furche and R. Ahlrichs, "Gaussian basis sets of quadruple zeta valence quality for atoms H–Kr," *J. Chem. Phys.* 119, no. 24 (2003): 12753. https://doi.org/10.1063/1.1627293.
[31] R. C. Sabando, C. Riplinger, F. Wennmohs, F. Neese and G. Bistoni, "Broadening the Scope of the ETS-NOCV Scheme: A Versatile Implementation in ORCA," *J. Chem. Theory Comput.* 21, no. 16 (2025): 7920. https://doi.org/10.1021/acs.jctc.5c01003.
[32] I. Raabe, K. Wagner and K. Guttsche et al., "Tetraalkylammonium salts of weakly coordinating aluminates: ionic liquids, materials for electrochemical applications and useful compounds for anion investigation," *Chem. Eur. J.* 15, no. 8 (2009): 1966. https://doi.org/10.1002/chem.200800417.
[33] "T. Janssen, A. Janner, A. Looijenga-Vos and P. M. de Wolff 2024, Incommensurate and commensurate modulated structures." in International Tables for Crystallography, edited by C. P. Brock, T. Hahn and H. Wondratschek et al., Chester, England: International Union of Crystallography. ISBN 978-1-4020-5259-0.
[34] A. Janner and T. Janssen, "Superspace groups," *Physica A* 99, no. 1-2 (1979): 47. https://doi.org/10.1016/0378-4371(79)90124-9.
[35] A. L. Spek, "Single-crystal structure validation with the program PLATON," *J. Appl. Crystallogr.* 36, no. 1 (2003): 7. https://doi.org/10.1107/S0021889802022112.
[36] M. Ligare, "Classical thermodynamics of particles in harmonic traps," *Am. J. Phys.* 78, no. 8 (2010): 815. https://doi.org/10.1119/1.3417868.
